# Time dynamics and invariant subnetwork structures in the world cereals trade network

**DOI:** 10.1371/journal.pone.0216318

**Published:** 2019-05-22

**Authors:** Marie-Cécile Dupas, José Halloy, Petros Chatzimpiros

**Affiliations:** Laboratoire Interdisciplinaire des Energies de Demain (LIED), Université Paris Diderot, Paris, France; University of Georgia, UNITED STATES

## Abstract

The development of industrial agriculture has enabled a sharp increase in food trade at the global scale. Worldwide trade underpins food security by distributing food surpluses to food deficient countries. The study of agricultural product flows can provide insights on the complex interactions between exporting and importing countries and the resulting network structures. Commercial partnerships between countries can be modelled using a complex network approach. Based on the detailed trade matrices from FAO covering the period from 1986 to 2013, we present an analysis of the world cereal trade in terms of weighted and directed networks. The network nodes are the countries and the links are the trades of agricultural products in mass. We reveal the changing topology and degree distribution of the world network during the studied period. We distinguish three entangled subnetwork structures when considering the temporal stability of the trades. The three subnetworks display distinct properties and a differential contribution in total trade. Trades of uninterrupted activity over the 28-year study period compose the backbone network which accounts for two thirds of all traded mass and is scale-free. Inversely, two thirds of the trades only have one or two consecutive years of activity and define the transient subnetwork which displays random growth and accounts for very little traded mass. The trades of intermediate duration display an exponential growth both in numbers and in traded mass and define the intermediate subnetwork. The topology of each subnetwork is a time invariant. The identification of invariant structures is a useful basis for developing prospective agri-food network modelling to assess their resilience to perturbations and shocks.

## Introduction

The outbreak of large-scale agricultural trade networks is a turning point in history. Advances in technology during the 19th century laid the basis of a rapid growth in long-distance trade triggered by the 1950s by agricultural intensification [1] [2] and decreasing transportation costs [3]. In particular, increasing grain production in Northern America and Europe by the 60’s opened the way to the integration of numerous new markets largely contributing to trade globalization [4]. Today, about 5.1 billion people are estimated to live in a net food importing country [5] and about 23% of total agricultural production is subject to international trade [6]. Cereals are the most traded agricultural products with 50% total mass traded (FAOstat [7], Food and Agriculture Organization) and account for more than half of the global average direct human intake [8] with 1292kcal/cap/day and for two thirds of the feed intake of livestock with 873 million tons (FAOstat [7]).

Climate change including warming, drought and weather extremes is highly likely to impact agricultural yields [9] [10] [11] and, thereby, the distribution of food availability. Because trade plays a key role in reallocating food production [12], addressing food security issues requires understanding the structure of trade networks. Depending on the structure, perturbing shocks will have different effects on the total network. Graph mathematical theory is a network science modelling approach that studies the graphs made by the nodes and links identified as relevant for describing a system. It defines metrics to capture the topology of empirical networks and tackle complex patterns of change [13] [14] [15]. The topology in graph theory describes the arrangement of nodes and links in networks and gives important information on the pattern of nodes connectivity. Here, we study the world cereal trade network, including all grain and grain-derived products based on the FAOstat database (1986-2013) and use the countries as nodes and the mass of international cereals flows as links.

Network science has been previously applied on food trade for several commodities at various scales and time points but little focus has been given on the complex time dynamics of networks. A branch of literature assesses the dependency of countries on food trade by quantifying net imports and exports of agricultural products [6] [5]. These studies do not intend to explicit food trade topology but provide insights on worldwide food availability and distribution [16], rarely on a dynamical basis [17]. Other studies focus on network properties at a single date or a limited set of years, providing a static view of the network topology and its basic metrics. Such studies have mainly focused on the connectivity structure of regional ([18] [19] [20]) or world trade for a set of agricultural products in terms of virtual water equivalent [21] [22] [23], land acquisition [24] or monetary volumes [25]. Other studies have identified and analyzed the community structures of different food product categories in terms of virtual water [26] and through multi-layer food networks [27].

Dynamical views on the world food trade network are less explored. A handful of studies have recently analyzed the changing topology of the world food network for seafood [28] and for several other commodities aggregated in terms of virtual water [29] [30] [31] [32] but the results lack a comprehensive analysis of the changing network properties. They reveal the dynamical behaviour of trades [29] and report a time-variable network structure by examining the network as a whole. Network evolution can however embed underlying structural invariants which attest for specific networks dynamics. Their detection is precious in complex system modelling for capturing prospective network evolution. Here, we explore the topology and changing properties of the world cereals trade network by detecting distinct trade substructures according to the trades temporal stability and reveal their structural invariants. The observed features are mainly driven by techno-economical and geopolitical factors.

## Main contributions

We show that the world cereal network has a topology that changes during the studied period (1986-2013). It transforms progressively from a scale-free network to an exponential degree distribution network. However, by considering the time duration of the trades, we reveal the existence of three entangled subnetwork structures with invariant topology. Only the parameters of the topology may change in time but not their nature.

We present the topology of each subnetwork and the evolution of their properties. Most of the trades are ephemeral with only one or two consecutive years of activity but contribute very little to the total mass traded. They constitute what we call the “transient” subnetwork. The trades that contribute the most in total traded mass have no interruptions during the studied period. They constitute what we call the “backbone” subnetwork. Trades that are neither transient nor continuous during the studied period display an exponential degree distribution and form what we call the “intermediate” network. The increasing number of intermediate trades changed the topology of the total network from a scale-free to an exponential degree distribution with increasingly small-world properties. The identification of these three invariant structures contribute to build prospective complex system modelling of food networks.

The singular structure of the backbone subnetwork shows the dominance of few occidental countries, the hubs, which are either large cereals producers or key redistribution countries of the world food resources thanks to their geographical position and powerful infrastructures. However, the emergence of the intermediate network reflects the increasing contribution of new actors in world cereal trade. These new actors can increase cereals availability and improve food security. The decreasing dominance of hubs in total trade could make the world network less vulnerable to local crisis.

## Materials and methods

### Data description

We analyze the world cereals trade network for the period covering 28 years from 1986 to 2013 based on the FAOstat detailed trade matrices [7]. The datasets provide the product flow quantities per origin and destination by country. Each country reports the traded quantity with every partner country. Hence, there are two reported values per trade (one of the importing country and one of the exporting country) which are not always in agreement. We built a unique trade matrix by averaging the values reported by the trading partners. In the case of missing data on behalf of one country, we considered the trade value reported by the partner country. We provide a detailed assessment of the data discrepancies in the supporting information (Part D in S1 Appendix). The resulting trade matrix is a bi-directional set of flows, meaning that trades of opposite directions do not cancel out. Each country can export to another country a product that it also imports from them.

The list of countries and territories in FAOstat varies in time due to national frontier changes. The number of nodes increased in 1991 from 200 to 221 nodes mainly due to the dissolution of the Soviet Union (Table C in S1 Appendix). Changes also concern several islands which change from independent territories to parts of mainland. Almost all countries are part of the network throughout the study period with at least one trade per year. The number of trades almost tripled from 3265 in 1986 to 8700 in 2013.

#### Products

We study the case of cereals which are the most traded products worldwide and constitute the basis of human diets. At the global scale, cereals represent around 1000 kcal/capita/day that is 50% of the daily diet per capita [33]. We aggregated data on cereals trade for wheat, rice, barley, maize, millet, fonio, buckwheat, sorghum, rye and oats considering both grain and product derivates. Overall, the matrices comprise 61 commodities out of which 15 are primary products and 46 are secondary products (Table A in S1 Appendix).

### Network description

A graph G is a pair of sets (V, E), where V is the set of nodes (*i.e*. the countries) and E is the set of links (*i.e*. the traded mass) between two nodes. Each country is labelled by its ISO code (Table B in S2 Appendix). The graph is described by a square matrix called adjacency matrix *A*. *A*_*ij*_ is equal to one if a trade exists between two nodes *i* and *j* and is zero if there is no trade between the nodes.

We describe the cereals trade network as a directed graph, therefore the matrix *A* is asymmetric and directional. The cereals trade network is weighted, therefore, the matrix *W* is a square matrix where *W*_*ij*_ is equal to the traded mass (metric tons) between nodes *i* and *j*. The resulting graph is a bi-directed network, *i.e*. a node *i* can have an import from a country *j*, and also an export to the country *j*. Hence, matrices *A* and *W* do not sum up. We consider individually all import and export trades (*i.e*. every physical flow) between two countries. The diagonal of matrices *A* and *W* are zero (trades from a node to itself).

### Definition of subnetworks: Backbone, intermediate and transient networks

We analyze the cereals trades according to their temporal stability. A trade between two countries can appear in several years. For each trade we determine the number of years of continuous activity. We define three categories.

Firstly, the trades which are active throughout the study period (28 years) are considered as permanent and compose the **backbone subnetwork**. The backbone subnetwork includes, in addition, the trades resulting from the break-up or merger of countries, provided that these trades remain active throughout the period. As an example, this is mainly the case of the dissolution of the USSR and the emergence of 15 new nodes in Europe by 1992. The trades that resulted from this dissolution are included in the backbone subnetwork if they remain active from 1992 onward and if a trade between the partner country and the USSR existed before 1992. Secondly, the trades which are active for only 1 or 2 consecutive years, are considered as ephemeral and are defined as the **transient subnetwork**. Thirdly, the trades which are active between 3 and 27 consecutive years are considered as intermediate and compose the **intermediate subnetwork**.

### Network analysis

We analyze the basic metrics of the cereals trade network such as network density, average path length, degree distribution, assortativity coefficient, centrality coefficients, clustering coefficients as defined in the graph theory [14] [34]. The network density is the probability that two randomly chosen nodes in the network are connected. If the density is equal to 1, all nodes are connected, and if it is zero there is no network.

The node degree is the number of trading partners of a node and defines the node connectivity. In a directed network, the node degree is composed by the in-degree *k*_*in*_ (*i.e*. the number of imports of a country) and the out-degree *k*_*out*_ (*i.e*. the number of exports of a country). The degree distribution is the probability that a node has *k* connections. The distributions are fitted using the powerlaw library [35] in Python. The parameters *α* and *k*_*min*_ of the power law function are those that minimize the Kolmogorov-Smirnov distance *D*_*n*_ between the data and the fit. The network is weighted by the mass traded per link to define the node strength. The node strength is the total mass imported and exported per country. The in-strength of a node is the total imported mass and the out-strength is the total exported mass. The connectivity distribution of nodes is shown for an unweighted network as the degree distribution *P*(*k*). The degree distribution *P*(*k*) represents the fraction of nodes with a number of degree equal to *k*. The cumulative density function *P*(*K* > *k*) of the network is the fraction of nodes with a number of degree higher than *k*.

Two additional measures to capture the connectivity between two nodes are the assortativity coefficient and the neighbor connectivity [36] [34]. The assortativity coefficient is the metric that corresponds to the Pearson correlation of the degrees of each pair of nodes. The value of this coefficient is between −1 and 1. A negative value means that the network is disassortative, and a positive value that the network is assortative. The neighbor connectivity is the average degree of the nearest neighbours of a given node, defined as:

$$\begin{array}{c} k_{nn,i}=\frac{1}{k_{i}}\sum_{j\in V_{i}}k_{j} \end{array}$$ (1)

with *k*_*i*_ the degree of the node *i* and *V*_*i*_ the set of neighbors of the node *i*. In an assortative network, the degree of nearest neighbour *k*_*nn*_ follows an increasing function of the node degree *k*. Conversely, disassortative networks have a decrease in the degree of nearest neighbour *k*_*nn*_ in function of the node degree *k*.

To find the triads that are representative of a directed network, we used four clustering coefficients. Each coefficient is representative of a triad and is comprised between 0 and 1. If the coefficient is 0 then there is no such triad in the network. The motifs are defined by Fagiolo [37] considering both weighted or unweighted networks. For a node *i*, the “In” coefficient *C*_*in*_ represents the probability that import neighbors are connected with each other; by analogy, the “Out” coefficient *C*_*out*_ represents the probability that export neighbors are connected with each other. These two coefficients do not consider the direction of flows between the two partners.

The *C*_*in*_ and *C*_*out*_ are defined as follows:

$$\begin{array}{c} C_{i}^{in}=\sum_{j,h\in V_{i}^{in}}\frac{\left(A_{ji}+A_{hi}\right)A_{jh|hi}}{2\times k_{i}^{in}\left(k_{i}^{in}-1\right)} \end{array}$$ (2)

$$\begin{array}{c} C_{i}^{out}=\sum_{j,h\in V_{i}^{out}}\frac{\left(A_{ji}+A_{hi}\right)A_{jh|hi}}{2\times k_{i}^{out}\left(k_{i}^{out}-1\right)} \end{array}$$ (3)

where A is the unweighted matrix of the graph, $k_{i}^{in}$ is the in-degree of the node *i*, $k_{i}^{out}$ is the out-degree of the node *i*, $V_{i}^{in}$ is the set of neighbors from which node *i* imports, and $V_{i}^{out}$ is the set of neighbors from which node *i* exports. We computed these coefficients for an unweighted directed network with Python and Networkx package [38].

The shortest path length is the minimum number of steps required to go from a given node to another node randomly chosen in the network. In directed networks, it is not always possible to define the shortest path for any pair of node. For instance, for isolated clusters, the shortest path length is infinite. Infinite path lengths are not taken into account in the calculation of the average path length of the network. In 1986, 27% of the pairs of nodes have a infinite path length against 11% in 2013.

The measure of node centrality can be calculated by several algorithms. We focus on the shortest path property of nodes by quantifying the betweenness centrality and the closeness centrality for the directed network. The betweenness centrality is the ratio between the number of shortest paths that go across a node and the sum of all shortest paths. The closeness centrality is the inverse of the sum of distances of the shortest paths that go across a node. The sum is normalized by the sum of the minimum possible distances *n* − 1.

## Results

### General evolution patterns of the world cereals network

The density of the total world cereals network has increased over the study period as a result of the increase in the number of trades with a constant number of nodes. In 1986, almost all countries were already active nodes in the network with an average of 34 trades per node, compared with 83 in 2013. This translates into a normalized density of 8.8% in 1986 against 19.8% in 2013 compared to a theoretical density of 1 in a network where all nodes are directly connected with each other. Accordingly, one fifth of the theoretical number of links (*n*^2^ − 1) were present in 2013.

The total traded mass more than doubled from 186 million tons in 1986 to 405 million tons in 2013, but the network density increased even faster. As a result, the average weight of links decreased faster than the average path length of trades, meaning that the world network is increasingly displaying “small-world” properties [39]. Small-world are networks where nodes can reach other nodes with a very few steps. In the world cereals trade network, any node of the network can be reached through an average of 1.8 steps in 2013, against 2.1 in 1986.

**Fig 1:**
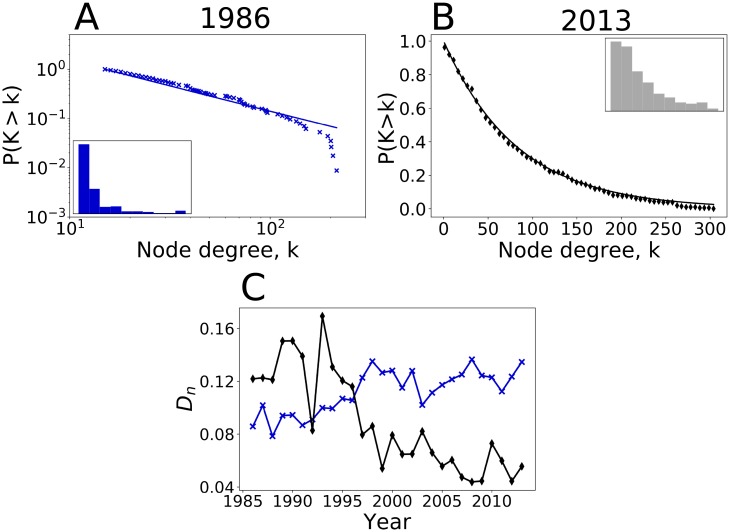
Evolution of the degree distribution of the world cereals trade network. **A. Degree distribution in 1986**. The cumulative density function follows a power law of type *P*(*K* > *k*) ∝ *k*^−*α*^) with *k*_*min*_ = 15 and *α* = 2.02. The Kolmogorov-Smirnov statistic is: *D*_*n*_ = 0.0857. The power law property is a characteristic of a scale free network, the degree of a few nodes greatly exceeds the average degree of nodes. The highest-degree nodes are the “hubs”, and serve specific purposes in the networks, here they are major exporting countries. **B. Degree distribution in 2013**. The cumulative density function fits with an exponential function of type $P\left(K>k\right)\propto \text{exp}\left(-\frac{k}{<k>}\right)$ where < *k* > = 82.85 is the average degree of nodes. **C. Kolmogorov-Smirnov distance**. The distance, *D*_*n*_, is represented over time for the fit with a power law distribution (blue line) and with an exponential distribution (black line). The Kolmogorov-Smirnov statistic, *D*_*n*_, gives the distance between the data and the fit. Minimizing this value equates to optimizing the fit. Until 1996, the *D*_*n*_ value was lower for the power law fit than for the exponential fit; the inverse is true after 1997. Thus, the fit of the power law becomes less accurate and the fit of the exponential distribution becomes more accurate. These two trends confirm the evolution of the degree distribution from scale free to exponential.

The topology of the total network has changed. Fig 1 shows that the node degree distribution in the total network followed a power law distribution in 1986 and an exponential distribution in 2013. The topology transition is shown by the increase in the Kolmogorov Smirnov distance of the power law fit and the decrease in the Kolmogorov Smirnov distance of the exponential fit (Fig 1C). The Kolmogorov Smirnov test is a statistic metric that computes the distance between the fit and the data. The parameters of the fitted functions are optimized by minimizing this distance. Thus, over time, the fit of the power law becomes less accurate and the fit of the exponential distribution becomes more accurate. The power law distribution (Fig 1A) is specific to a scale-free network.

The topology transition from a scale-free to an exponential distribution (Fig 1B) highlights that the links added after 1986 did not follow a model of preferential attachment. This transition is due to the emergence of new powerful nodes, mostly corresponding to developing countries which agricultural potential has previously been largely under-exploited such as Turkey, India and Malaysia. In these three countries, the cereal yields doubled since 1986 (Figs N10 & N11 & N12 in S4 Appendix).

### Decomposition of the world cereals network according to the trades duration

**Fig 2:**
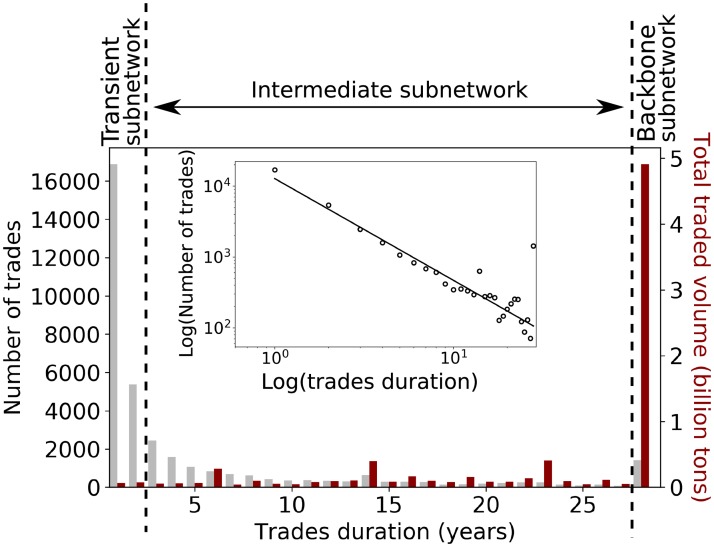
Distributions of the trades duration and their associated traded mass. The distribution of the number of trades (grey) and their associated traded quantities (red) is represented as a function of the number of consecutive years of activity in the period from 1986 to 2013 (28 years). The inset figure shows that, for the world network, the distribution of the number of trades over time fits with a power law with an alpha parameter equal to −1.44 and a correlation coefficient equal to 0.98. According to the duration distribution of trades we can distinguish three zones. The first zone corresponds to trades of 1 or 2 consecutive years of activity and define the **transient subnetwork**. The second zone corresponds to trades of 3 to 27 consecutive years of activity and define the **intermediate subnetwork**. The third zone corresponds to trades that are continuous throughout the 28-years study period and define the **backbone network**. Most of the traded mass (bars in red) belongs to the backbone subnetwork.

Fig 2 shows the distribution of total trades and their associated mass according to the trade duration. The duration distribution follows a power-law relationship highlighting the high heterogeneity in the trades temporal stability as seen in the inset of Fig 2. Considering the differences in the continuous duration among trades, we can divide the total network into three subnetworks with distinct properties and dynamics (see Sec. Materials and methods). In contrast to previous studies, we show that rather than a single network structure under change, the world cereals trade network is a composite structure resulting from the entanglement of three subnetworks, each one characterized by a distinct and time-invariant topology.

**Fig 3:**
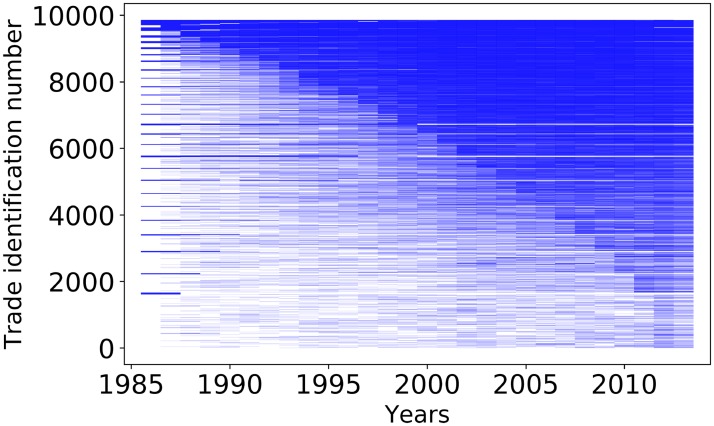
Time series of trades in the intermediate subnetwork. This graph shows the activity for the 9858 trade exchanges belonging to the intermediate subnetwork *i.e*. trades with duration comprised between 3 and 27 consecutive years. If the blue line is continuous then the trade has no interruption. Trades are sorted according to their cumulative lifetime starting from the shortest cumulative periods. The distribution of the continuous duration of trades is heterogeneous and only a minority of trades last during the maximum duration of 27 consecutive years.

**Fig 4:**
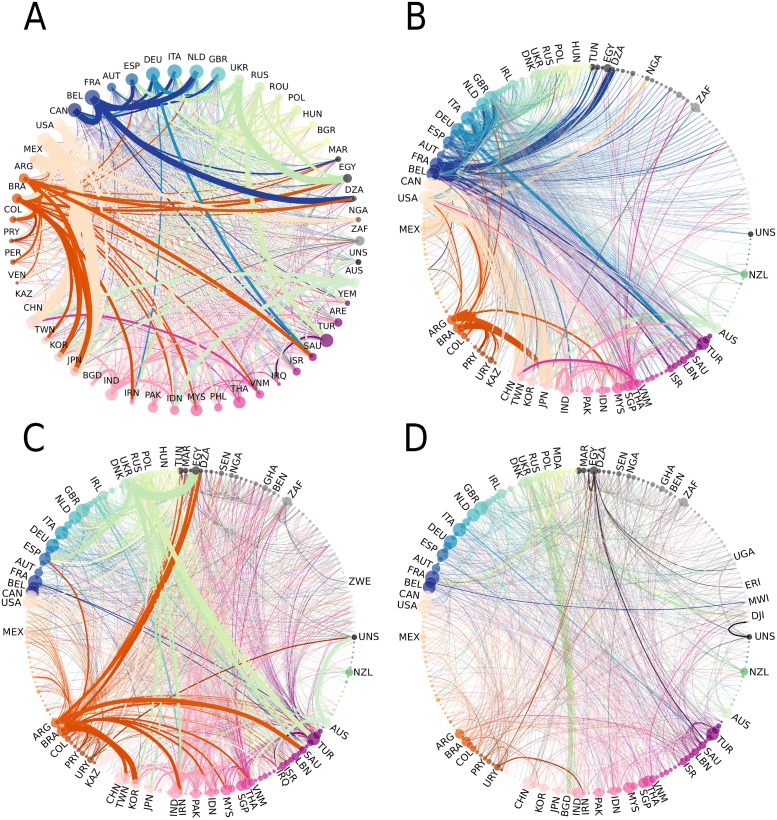
The total world cereals trade network and its decomposition in the three subnetworks in 2013. The countries are ranked by continent and the size of nodes is weighted by the total number of trades *k*_*tot*_. The color code indicates the continent group: grey, Africa, green Oceania, blue to yellow Europe, orange to red America, pink Asia. The edges are weighted by the traded mass and the colour corresponds to the country of destination. **A. World cereal network**. The graph is limited to the 50 most important countries and their trades. The total network counts 210 nodes and 8700 links. The total traded mass is 405.7 million tons. The degree distribution is of type exponential. **B. Backbone subnetwork**. This network counts 202 nodes and 2135 links. The traded mass is 276 million tons, which represents 68% of the world trade in 2013. The degree distribution follows a power law indicating that the network is scale-free. **C. Intermediate subnetwork**. This network counts 208 nodes and 4685 links. The traded mass is 121 million tons, which represents 29% of total trade in 2013. The degree distribution is of type exponential. **D. Transient subnetwork**. This network counts 207 nodes and 1880 links. The traded mass is 8 million tons, which represents about 2% of total trade in 2013. The degree distribution is of type exponential.

The **transient subnetwork**, comprising trades of short duration *i.e. 1 or 2 years*, gathers about two thirds of all trades but only accounts for 1.7% of the cumulative traded mass over the study period. The trades with a duration of between 3 and 27 years define the **intermediate subnetwork**. The stability of the intermediate trades increases over time (Fig 3). The intermediate subnetwork accounts for 36% of the cumulative traded mass over the study period. The third and last trade class contains the uninterrupted trades throughout the study period (trades lasting for 28 years or more). This class defines the **backbone subnetwork** and accounts for 62% of the cumulative traded mass. Carr *et al*. [29] have also defined a backbone food subnetwork as the trades that make up 80% of the total traded mass. The backbone subnetwork defined here points out the long-lasting commercial partnerships worldwide. The three subnetworks are visualized in Fig 4.

### Distinct structural properties of the three subnetworks

#### General metrics of each subnetwork

**Fig 5:**
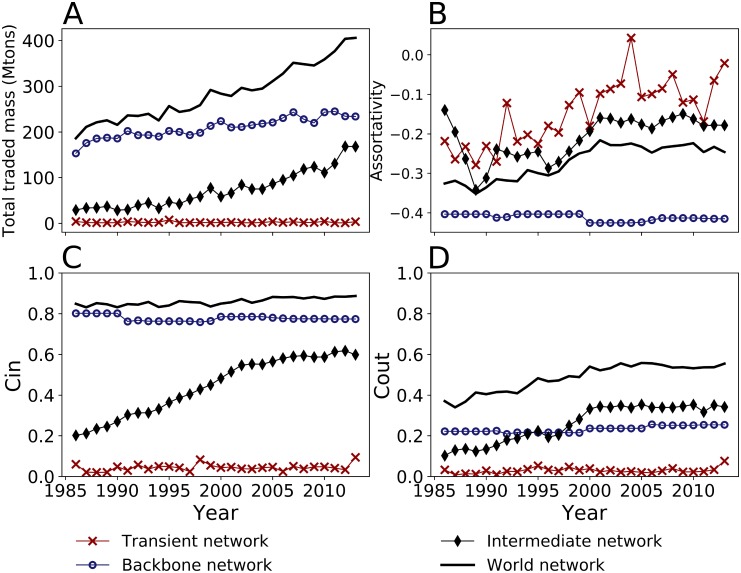
Evolution of the metrics of the total cereal network and the three subnetworks. A. Total traded mass. B. Assortativity coefficient. C. Clustering coefficient of type In. D. Clustering coefficient of type Out.

Fig 5A shows the traded mass per subnetwork and the total traded mass over time. From the three subnetworks, the backbone subnetwork accounts for most of the traded mass both in cumulative terms and per year, with an average annual traded mass of 234 million tons and a steady annual increase of about 2.25 million tons/yr. However, its share of the world traded mass decreased over time in favor of the intermediate subnetwork. In 1986, the backbone subnetwork accounts for 83% of all traded mass, and in 2013 it accounts for 58% (Fig F in S1 Appendix).

The intermediate subnetwork is an emerging structure. It includes trades which stability increases over time. It is the one that grew the most significantly during the study period, from about 1047 links in 1986 to 5422 links in 2013 (Fig F in S1 Appendix). The traded mass increased even more rapidly from 29 million tons in 1986 to 168 million tons in 2013 (Fig 5A) accounting respectively for 15% and 42% of all cereals trade (Fig F in S1 Appendix). In 2013, the largest intermediate trades are from Ukraine to Egypt (5.97 million tons), from Brazil to the Republic of Korea (3.90 million tons) and from Russia to Turkey (3.50 million tons). The average mass of trades is 31035 tons and the median value is 221 tons which are sensibly lower than the trades in the backbone network with an average mass of 113106 tons and a median value of 3586 tons.

The transient subnetwork is defined by trades that only last 1 or 2 consecutive years. Transient trades are very numerous but very weak in mass both in 1986 and 2013 with respectively 4 and 3.5 million tons (Fig 5A). Nonetheless, the number of trades doubled from 597 in 1986 to 1210 in 2013 (Fig F in S1 Appendix). This doubling implies high economic opportunities for trading low cereals mass. The share of the transient subnetwork in total traded mass dropped from 2.1% to 0.8% (Fig F in S1 Appendix). In addition, the average mass of trades is 2920 tons and the median value is 15 tons in 2013.

#### Invariant topology of the three subnetworks

- Node degree distribution.

**Fig 6:**
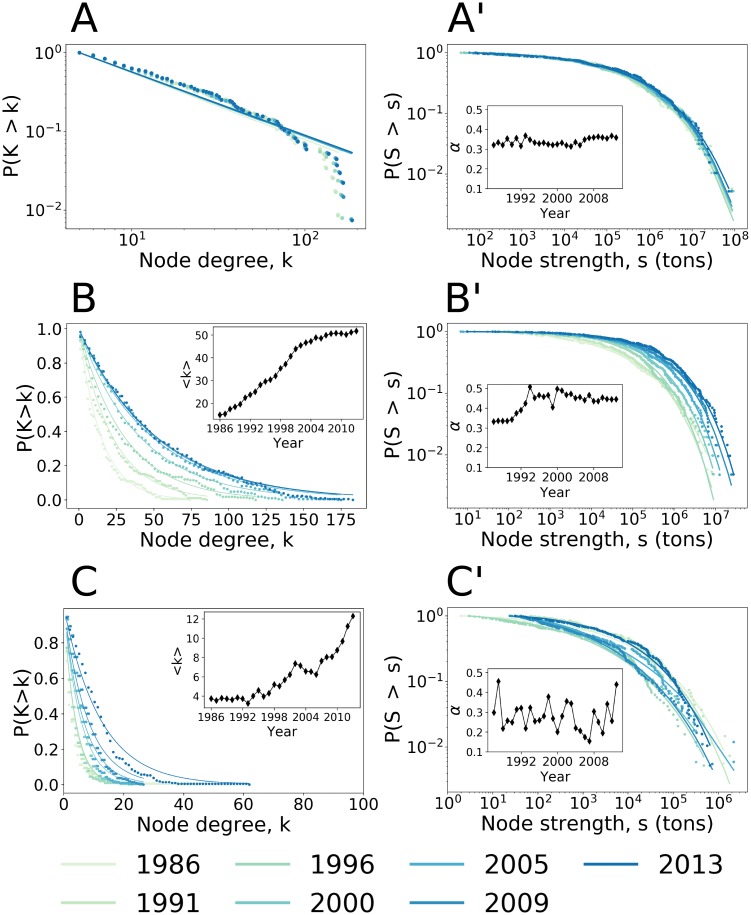
A, B, C: Degree distribution of the three subnetworks. **A’, B’, C’: Node strength distribution of the three subnetworks**. The distribution of the nodes strength follows a stretched exponential function of type *P*(*K* > *k*) = exp((−λ*k*)^*α*^). The inset figures show the evolution of the average degree < *k* > (B and C) and the *α* parameter evolution of the stretched exponential equation (A’,B’,C’). **A. Degree distribution of the backbone subnetwork**. The degree distribution follows a power law of type *P*(*K* > *k*) ∝ *k*^−*α*^). The values for 2013 are *k*_*min*_ = 7 and *α* = 1.83 and the Kolmogorov-Smirnov statistic is: *D*_*n*_ = 0.077. As the *α* parameter is less than 2.0, the standard deviation and the mean (< *k* >) are undefined. The hubs are Belgium, USA, France, The Netherlands, Italy, Germany and United Kingdom. **A’. Node strength distribution of the backbone subnetwork**. The stretched exponential distribution and its *α* parameter are stable in time. **B. Degree distribution of the intermediate subnetwork**. The cumulative density function fits with an exponential function of type $P\left(K>k\right)\propto \text{exp}\left(-\frac{k}{<k>}\right)$. In 2013, the average nodes degree is < *k* > = 51.67 and the correlation coefficient is *R*^2^ = 0.99. **B’. Node strength distribution of the intermediate subnetwork**. The stretched exponential distribution changes over time as shown by the decrease and stabilization of the *α* parameter. **C. Degree distribution of the transient subnetwork**. The degree distribution of the transient subnetwork fits with an exponential function. In 2013, the average node degree is < *k* > = 11.98. The correlation coefficient is *R*^2^ = 0.97. **C’. Node strength distribution of the transient subnetwork**. The evolution in time of the stretched exponential distribution is erratic as shown by the random changes in the *α* parameter.

The degree distribution (for a definition see Sec. Materials and methods) of each subnetwork is shown by the cumulative density function of the nodes degree and differs among the three subnetworks and the total network (Fig 6A, 6B & 6C). For the backbone subnetwork (Fig 6A), the cumulative density function of the degree distribution is fitted with a power law function *P*(*K* > *k*) ∝ *k*^−*α*^). The scaling property highlights that no particular scale characterizes the system. Only a few nodes are highly connected, the “hubs”, and the majority of peripheral nodes are preferentially connected to them.

The degree distribution of the intermediate subnetwork is of type exponential [40] $P\left(K>k\right)\propto \exp\left(-\frac{k}{<k>}\right)$ with average degree of nodes of < *k* > = 51.67 in 2013. This type of graph can correspond to a random growth throughout the study period indicating no preferential attachment of trades to nodes. The growth of the intermediate subnetwork has driven the observed densification and the change in the degree distribution of the total network from a power law function in 1986 to an exponential function in 2013.

The transient subnetwork has a random structure and displays no preferential attachment to highly connected nodes. The amplitude of the node degrees is quite narrow (0-60 degrees) indicating a rather uniform degree distribution (Fig 6C’). The average node degree is of 6.67 links per node in 1986 against 11.98 links per node in 2013. This subnetwork most likely captures the existence of random but still profitable commercial opportunities for trading little mass of cereals over long-distances based on low-cost transportation.

- Assortativity analysis.

The assortativity coefficient (Fig 5B) (for a definition see Sec. Materials and methods) of the backbone subnetwork is negative, meaning that nodes attach preferentially to nodes of different connectivity and that only a few nodes, the hubs, are highly connected. The disassortativity property relates to the scale-free structure [41] observed with the degree distribution. It is also highlighted by the fact that the neighbours of hubs in the backbone subnetwork have low node degrees. This features is stable in time (Fig J in S2 Appendix).

In the intermediate subnetwork, the assortativity coefficient is higher than in the backbone subnetwork, indicating that the intermediate subnetwork is more homogeneous in terms of connectivity, *i.e*. there is less differentiation between highly connected and poorly connected nodes. The assortativity of the intermediate subnetwork increased in time. In 1986, the neighbours degree decreased with the node degree as opposed to 2013 where the nearest neighbours degree is relatively constant (Fig J in S2 Appendix).

The high volatility in the topology of the transient subnetwork is highlighted by the high fluctuations of the assortativity coefficient (Fig 5B). The nearest neighbours degree fluctuates with no clear tendency with the node degree throughout the study period highlighting the random behaviour of the transient subnetwork (Fig J in S2 Appendix.

- Node strength distribution.

The node strength distribution of the three subnetworks (Fig 6A’, 6B’ and 6C’) corresponds to a stretched exponential function *P*(*K* > *k*) = exp((−λ*k*)^*α*^) as Konar *et al*. found for the world virtual water network [32] [21]. The stretched exponential function is curved in a log-log plots [42]. The *α* parameter gives important indication on the distribution: if *α* is equal to 1 then the distribution is exponential; if it is comprised between 0 and 1, the distribution is stretched. The time evolution of the parameter *α* is given in the insets of Fig 6. The nature of the node strength distribution is common among the three subnetworks but the distributions differ in terms of magnitude and evolution.

The backbone subnetwork is stable in terms of node strength distribution over the study period, as reflected by the small variation in the *α* parameter. From the three subnetworks, the backbone has the lowest *α* parameter, with a stable value between 0.31 and 0.35 over the study period, meaning that it has the most stretched distribution. We observe more changes in the node strength distribution of the intermediate subnetwork, where the *α* parameter grew from 0.34 in 1990 to 0.51 in 1994 and then stabilized. The increase in the *α* parameter indicates a progressive reduction in the stretch of the exponential distribution, equating to a progressive homogenization among countries with very low and very high trading mass. However, the most important nodes in terms of traded mass belong to the backbone subnetwork where the contrast between low and high trading nodes persists. The transient subnetwork confirms its random characteristics. The node distribution varies during the study period and has no defined pattern of evolution as noted by the erratic variation of the *α* parameter between 0.12 and 0.45 over the study period.

#### Centrality and connectivity of countries

Centrality measures at what extend a node belongs in the shortest path between two randomly chosen nodes in the graph. In other words, it measures how much a country works as an intermediary between two distant countries. Nodes with high centrality are influential and are also expected to be the highest connected nodes.

Hubs in the backbone subnetwork are defined by both high connectivity (*i.e*. number of trades per node) and centrality meaning that they connect many nodes among them and through the shortest paths. Hubs are mainly European countries: Belgium, France, The Netherlands, Italy and Germany, plus the United States which are the second largest exporter in terms of number of trades (Table D in S3 Appendix).

In the intermediate subnetwork, as opposed to the backbone subnetwork, the nodes with the highest connectivity do not have the highest centrality (Tables F & G & Fig M in S3 Appendix). High centrality nodes support trades that are important in the network to link highly and poorly connected nodes. Accordingly, hubs in the intermediary subnetwork are highly connected but relatively poorly involved in the shortest paths.

In 2013, the nodes with the highest connectivity in terms of number of trades in the intermediate subnetwork are Turkey, India and Malaysia (Table E in S3 Appendix). In terms of traded mass, high connectivity nodes are Ukraine, Brazil and Russia. In contrast, the nodes with the highest centrality coefficients (supporting the shortest paths) are the United States, France, Canada, and Great Britain. These nodes are already hubs in the backbone subnetwork.

In contrast to the hubs in the backbone subnetwork, hubs in the intermediate subnetwork change in time (Table E in S3 Appendix). In the period of 1986-1995, hubs are mainly historical industrial countries, mostly Western European countries and the USA. Since the late 1990’s, the intermediate subnetwork is being increasingly led by developing countries, mainly Asian and South American countries (Turkey, Malaysia, Pakistan, China Argentina and Brazil) as well as some Eastern European countries such as Poland, Ukraine and Russia.

Another way to understand the emerging dynamics of the intermediate subnetwork is to compare the evolution of node connectivity with that of the backbone subnetwork. The node degree between the two subnetworks relate through a logistic function (Fig K in S2 Appendix). Node connectivity increases rapidly in the intermediate subnetwork and there are less and less nodes with lower connectivity than in the backbone subnetwork. Since 2000, only the five major hubs have conserved a higher connectivity in the backbone than in the intermediary subnetwork: Belgium, France, the United States, the Netherlands and Italy.

#### Clustering analysis

It is possible to measure the clustering of a node with its partners by taking into account the direction of flows. This can be done by the clustering coefficients. The analysis consists to statistically determine the most likely flow directions among three nodes at a local scale and average them at the entire network. The average clustering coefficients for a directed network allow identifying the existence of triads (Fig 5C & 5D).

The clustering coefficients that are the most representative in the network are of type ‘In’ and ‘Out’. The average clustering coefficient *In* and *Out* is higher in the three subnetworks than in an equivalent random network computed for each case, meaning that the three subnetworks are clustered. The most representative triad is of type *In* in both the total network and the backbone subnetwork. When a node has two imports from two different countries, the probability that these countries also exchange directly is high. The fact that the *In* coefficient is very similar between the total network and the backbone subnetwork means that the backbone subnetwork largely determines the clustering of the total network.

By analogy, the *Out* coefficient represents the probability that the neighbors of an exporting node also exchange directly between them. In the total network, the clustering coefficient of type *Out* is lower than the *In*, confirming the dominance of large exporters. A higher *In* than *Out* coefficient means that two exporting countries are more probable to trade directly between them than two importing countries. In the intermediate subnetwork, the probability that the partners of an exporting country have a trade between them increased over time and led to a higher *Out* coefficient in the total network.

#### Geographical analysis

**Fig 7:**
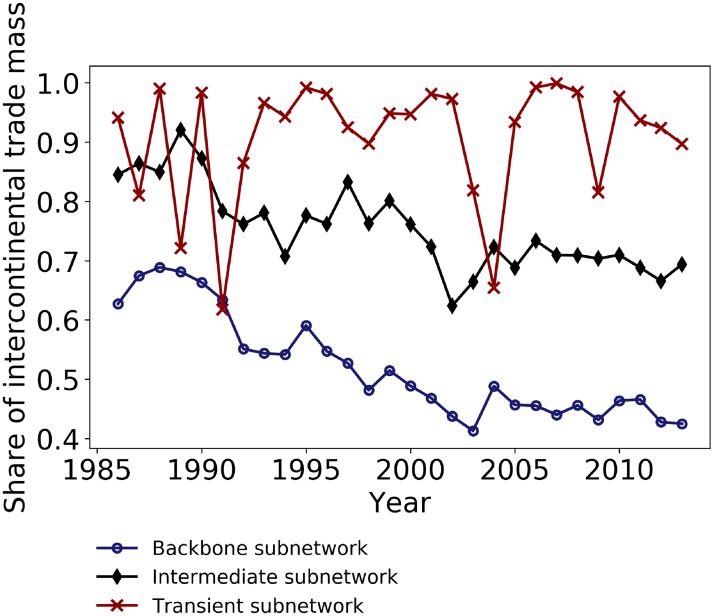
Share of intercontinental traded mass. The share is the ratio of intercontinental to total traded mass for each subnetwork. The transient subnetwork (blue dashed line) is mainly composed by intercontinental trades. In contrast, the share of intercontinental traded mass is being decreasing in time for both the intermediate subnetwork (red line) and the backbone subnetwork (black line). The backbone subnetwork is the least intercontinental subnetwork over the entire study period.

In addition to differences in the network topology and connectivity, the three subnetworks also differ in terms of geographical reach of trades. To characterize geographic distances between two partner countries, we count the number of intercontinental trades (Fig 7). In 2013, 43% of the mass traded by the backbone subnetwork was intercontinental against 57% between countries of a same continent. The backbone subnetwork is the least intercontinental subnetwork over the entire study period. Nevertheless, two among the largest trades in the backbone subnetwork are intercontinental: from the United States to Japan (10.2 million tons) and to China (8.6 million tons). The United States also have important neighbouring partners as they import 8.2 million tons of cereals from Canada and export 11.9 million tons of cereals to Mexico. The third largest intercontinental trade in 2013 is from France to Algeria (4.9 million tons).

The continent where the backbone subnetwork is the most developed is Europe with 107 trades between 27 countries and a traded mass of 12.4 million tons. The largest flows are exports from France to Belgium (5.2 million tons) and to the Netherlands (4.3 million tons). The long-lasting trade activity in Europe—represented by the backbone subnetwork—is supported on the one hand by geographic proximity and geopolitical and economic treaties that favor commercial stability and on the other hand by reliable production. Cereals production in Europe, and particularly wheat production, has increased fourfold since the 1960’s and varies little, potentially due to the adoption of the Common Agricultural Policy giving way to the intensification of trade within Europe and the competition with the United States for reaching external markets [4].

In contrast to the backbone subnetwork, the large majority of transient trades are intercontinental. In 2013, 90% of the transient trades were intercontinental even though these trades contribute to only 1% of total intercontinental traded volume. Similarly, intercontinental trades are dominant in the intermediate subnetwork, even though their share decreased over time. The share amounted to 85% in 1986 against 70% in 2013 besides the five-fold increase in the traded mass by this subnetwork. The decreasing share of intercontinental trades suggest a more rapid intensification in trades within continents.

## Discussion and conclusion

This study provides a detailed analysis of the patterns and evolution of the world cereals trade network. It reveals the invariant sub-network structures that underlie the global network dynamics and evolution. The identification and analysis of three subnetwork structures with time invariant topology over a 28-years-long time-period provides relevant insights on metrics that are usually studied either statically or for the entire network [43] [21] [23] [24] [25] [44] [29].

It has been previously shown that the global network increasingly displays small-world properties, meaning that countries are more closely connected as a result of increasing network density [23] [21]. Small-world properties are here confirmed and emphasized by the increase in the clustering coefficients and the decrease in the shortest path length over the study period [39] [45]. We show that increasing small-world properties are driven by the emergence of trades with exponential degree distribution (intermediate subnetwork) corresponding to developing countries that become international trade actors either by better exploiting their agricultural potential, through better infrastructures, through changes in domestic demand or a combination of these factors. Such countries represent a growing share in total trade but their activity does not modify the scale-free structure of the backbone subnetwork composed by the historical long-lasting trades. The trades that are neither in the backbone nor in the intermediate subnetworks are transient and have random structure. The transient subnetwork also grew significantly since 1986 but only in the number of trades as opposed to total traded mass which remained quasi-stable. Transient trades introduce randomness in the total network. The great number of links (62% of all trades since 1986) highly influences the degree distribution of the global network when considered unweighted. Weighting according to the traded mass greatly lowers the influence of transient trades on the topology of the total network. The three subnetworks identified in the present study display distinct and time-invariant structures.

The change in the structure of the global network from scale-free to exponential between 1986 and 2013 implies that the growth in traded mass during this period did not follow a pattern of preferential attachment to the highest connected nodes. The conservation of the scale-free property within a network with a constant number of nodes is possible up to a certain threshold and would necessitate preferential attachment [46]. In the agri-food context, the number of nodes is constrained by the number of countries and therefore the network evolution merely depends on the node connections. Here we observe a random growth with exponential degree distribution. Random growth and its impact on the network properties is given little scrutiny in literature [40]. In the agri-food context, no preferential attachment equates to the multiplication of powerful agricultural nodes with exportable grain surpluses supporting the diversification of grain supply sources to deficit countries. Diversification leads to a more homogeneous degree distribution and decreases the distance between the average and the maximum degrees [40]. The exponential distribution is mainly supported by emerging countries and is also reported for networks with high connectance and high average degree, such as the Worldwide Marine Transportation Network [47], email networks [48], the electric power grid of Southern California [39] and some food webs [49].

In previous food trade network studies the results on degree distribution of the global network often diverge. Most studies show an exponential distribution of degrees [32] [29] [50] [31] but others report a fat tail distribution [18] [25] [24]. Konar *et al*. [21] showed an exponential degree distribution for the virtual water trade network, but the study concerns a single date *i.e*. 2000. Carr *et al*. [29] analyzed the evolution of the topology of the global network (1986-2008) and found that the degree distribution follows an exponential function for the entire study period with a tendency to become more homogeneous over time. Ercsey-Ravasz *et al*. [25] found a broad degree distribution for the year 2008 implying a fat tail distribution similar to the power law distributions that we found for the first years of the study period.

Possible reasons of disagreement among studies include the variety of the studied commodities, author’s assumptions, the use or not of bi-directional matrices (see Sec. Materials and methods) and the accounting unit considered. Indeed, network analysis is rarely based on the physical flows of commodities but more on one of their resource equivalents. Most studies use the virtual water equivalent of trades [31] [23] [30] [29] [21] [32]. The network topology when calculated on the basis of virtual water may diverge from the topology calculated on the basis of the physical flows of products because of the differential water intensity in agricultural production of countries. The water intensity varies among countries according to production practices and climatic conditions. The physical flows of products are more relevant to describe the topology of the food network.

The decreasing heterogeneity between high and low connected nodes is a fundamental change in the topology of the world network. It is highlighted both by the change in the nodes degree and by the increase in the assortativity coefficient over time. Previous works focusing at a specific date reported a high disassortativity coefficient [21] [29] and an exponential degree distributions of node connectivity at several scales [20]. We show that disassortativity and scaling decreased over time due to the expansion of the intermediate subnetwork. The parameters of the exponential function evolved and result in the increase in average degrees in the topology of the global network. Nonetheless, despite the change in the topology and the metrics of the global network, the topology of each of the three underlying subnetworks structures remained invariant.

The scale-free property is also found in the node strength distribution with a stretched exponential function for the three subnetworks. The node strength is the sum of the mass of imports and exports. The stretched exponential distribution presents a tail fatter than the exponential distribution but thinner than the power law distribution [42]. This characteristic implies that the strength of nodes in terms of total mass is more heterogeneous than the nodes degree distribution. This feature is confirmed by the power law relationship between the node strength and the node degree (Figs H & I in S2 Appendix) [21] [29] [30] and implies that the more a country has trades and the more these trades are of high mass.

The global food network is highly influenced by geopolitical and economic treaties. A spectacular growth rate of the intermediate subnetwork in terms of mass, 42%, occurs in 1995. In 1992, the Cairns Group of Fair Trading Nations advocated for the reduction in trade barriers for cereals in the world economy. Agriculture is then included in the General Agreement on Tariffs and Trade (GATT) replaced, in 1995, by the World Trade Organization. In 1995, the Agreement On Agriculture (AoA) took effect and aimed at liberalizing agricultural markets [4].

Some countries of the Cairns group have become hubs in the intermediate subnetwork such as Canada, Argentina, Brazil, Malaysia, South Africa and Pakistan (Tables F & G in S3 Appendix). It is clear that trade barriers have played an important role in the cereals trade network: prior to the AoA, the world cereals trade market was dominated by Europe and the United States (Table D in S3 Appendix). European countries have already engaged in the intensification of cereals production since the 60’s and Europe shifted from a net cereals importer to a net exporter in the 80’s [4]. The breakdown into subnetworks seems to be relevant from a geopolitical point of view: the backbone subnetwork is dominated by Europe and the United States and the intermediate subnetwork by new exporting countries of the Cairns Group. This hypothesis will have to be further analyzed in a future work.

The hubs of the backbone subnetwork are mostly developed countries that historically correspond to the largest cereal producers on each continent (Table D in S3 Appendix). Agriculture is heterogeneously distributed throughout the world and only a few countries are powerful producers and reliable exporters. However, some countries are hubs not because of they produce much but because they are commercial crossroads due to their geographic location and powerful infrastructure. Often, these countries have important ports that serve as platforms to international trade. Countries with chronic cereal deficits tend to trade with hubs to benefit from their stability and reliability.

France, Great Britain, Germany, Belgium and the Netherlands are the main hubs in the region of Europe. France has a production of 1.06 *tons*/*cap* in 2013 [7], which is one of the biggest worldwide. In contrast Belgium and the Netherlands are major hubs with exceptionally low production. Despite their high productivity in terms of tons/ha, Belgium and the Netherlands rank at the 60th and the 114th positions in terms of world production in 2013 with 0.10 *tons*/*cap* and 0.30 *tons*/*cap* respectively. Their status as hubs in the backbone subnetwork clearly relates to their geographical position and port infrastructures and highlights their key role and long-term stability as cereal trade re-exporters. On the one hand, the United States and Canada with respective cereal production of 1.38 and 1.88 *tons*/*cap* and on the other hand Argentina and Brazil are the hubs in the American continent and have a great trade influence on Eastern Asia and Oceania. In this latter region, the major local hub is Australia.

The backbone and intermediate subnetworks are entangled in terms of node centrality, which is the degree of node insertion in the shortest trading paths. Nodes with high centrality are influential and are also expected to be the nodes with the highest connectivity. The nodes with the highest centrality in the intermediate subnetwork are hubs in the backbone subnetwork: the United States, Canada, the United Kingdom and France. Due to their high centrality, those countries are important intermediary nodes despite a lower number of trades in the intermediate subnetwork (Table G in S3 Appendix). Therefore, the hubs in the backbone subnetwork are not the highest connected nodes in the intermediate subnetwork, but their trades are important paths of re-exportation.

The three subnetworks show significant differences in terms of intercontinental trades. Such differences underline different trading logic and are highlighted in this study in terms of geographical reach and stability of trades. The backbone subnetwork is the least intercontinental and relies mainly on land transport infrastructure that is less cost-effective than maritime transport. The trade stability reflected by this subnetwork seems to witness the positive effect of proximity on long-lasting partnerships and economic cooperation. In contrast, the intermediate and transient subnetworks have a vast majority of intercontinental trades accounting for most of the mass traded by these subnetworks (Fig 7). They both mainly build on permanent maritime and at a lesser degree air traffic infrastructures and highlight the increase in agri-food globalization. The intermediate subnetwork reflects the progressive consolidation of long-distance trade partnerships between countries with cereal deficits and surpluses that respectively accentuate with demographic growth and agricultural intensification over time. In contrast, the transient subnetwork is highly volatile. The observed patterns of instability could support the idea that trades in the transient subnetwork are occasional and highly responsive to small and unplanned grain shortfalls or surpluses worldwide. Another possibility is to associate transient trades to errors in the FAOstat dataset but this option is only probable when the trade is reported once. When the trade is reported by both the importing and exporting countries, which is the case of most trades (Fig A in S1 Appendix), the data are most likely consistent.

Probably the main interest in the analysis of network topology is to assess network resilience to perturbations [51]. Perturbations may be geo-politically driven or relate to natural mechanisms such as climate change. A scale-free network, shaped by the activity of hubs, is more resilient to random failure than an exponential degree distribution network but much more vulnerable to targeted attacks [14] or globally synchronized shocks [52]. In exponential networks, degree distribution and connectivity are more homogeneous and the role of hubs and the average path length are lower than in scale-free networks. As a result, other trade paths to compensate for the removal or damage of a major node are facilitated and underlie higher network resilience.

Resilience can be modelled by simulating shocks and their propagation within food networks, but studies on this topic do not reach a consensus on the world food network. Some argue that the increase in connectivity does not increase food security [53] while others assert that highly connected networks tend to gain in stability [43] [50]. The insights provided in this study on the existence and properties of the invariant subnetwork structures within the world trade network can improve prospective food security modelling. The identification of invariant structures allows breaking-down complexity according to differential dynamics and contribution of each subnetwork in total trade. The temporal stability of trades seems to relate to the geopolitical evolution of the world agricultural markets. The decomposition into invariant structures can, therefore, support complex system prospective modelling of food security accounting for climate change and geopolitical constraints based on simple criteria such as preferential attachment to hubs or random connectivity. The analysis of agricultural world trade from a perspective of distinct subnetworks could be extended to other key agricultural products such as feed, meat, fresh food and beverages to assess whether the detected invariant structures are specific to cereals or generic among all food commodities.

## Supporting information

S1 Appendix Data description and curation. (PDF)

S2 Appendix Connectivity of world cereals trade network. (PDF)

S3 Appendix Network centrality measures. (PDF)

S4 Appendix Characteristics of cereals production for some countries. (PDF)
