# Supplementary material for: Time dynamics and invariant subnetwork structures in the world cereals trade network
Source: PLoS One. 2019 May 22;14(5):e0216318. doi: 10.1371/journal.pone.0216318 (PMC6530828; doi:10.1371/journal.pone.0216318)
Supplement: S1 Appendix — (PDF) [file pone.0216318.s001.pdf]

Supporting information

S1 Appendix. Data description and curation

A List of cereal products

We have aggregated the cereal products available in the FAOstat database. The main traded cereals are Wheat, Rice, Barley and Maize. We also take into account the processed products of primary cereals. All the products studied are in the Table A.

Item name		Item code
Wheat		15
	Flour of wheat	16
	Macaroni	18
	Germ of wheat	19
	Bread	20
	Bulgur	21
	Pastry	22
	Starch of Wheat	23
	Wheat Gluten	24
Rice, paddy		27
	Rice, husked	28
	Rice, Milled	31
	Rice, Broken	32
	Rice, Gluten	33
	Starch of Rice	34
	Flour of Rice	38
	Breakfast Cereals	41
Barley		44
	Pot Barley	45
	Barley, Pearled	46
	Barley Flour and Grits	48
	Malt	49
	Malt Extract	50
Maize		56
	Germ of Maize	57
	Flour of Maize	58
	Maize Gluten	63
	Starch of Maize	64
	White Maize	67
	Popcorn	68
Rye		71
	Flour of Rye	72
Oats		75
	Oats, Rolled	76
Millet		79
	Flour of Millet	80
Sorghum		83
	Flour of Sorghum	84
Buckweat		89
	Flour of Buckwheat	90
Quinoa		92
Fonio		94
	Flour of Fonio	95
Triticale		97
	Flour of Triticale	98
Canary seed		101
Mixed grain		103
	Flour of Mixed grain	104
Cereals, nes		108
	Infant food	109
	Wafers	110
	Flour of Cereals nes	111
	Cereal Preparations	113
	Mixes and Doughs	114
	Food Preparations of Flour	115

Table A. List of cereals products in the FAOstat database. The first column contains the primary products, the second column the secondary products and the third the item code.

B List of countries

Each country is associated with an ISO code (3 letters) for visualization, and a color range according to the continent. The list of countries can be found in the following Table B.

Code Color	Continent	Country code in FAO	Country	ISO code
1	Africa	24	British Indian Ocean Territory	IOT
1	Africa	29	Burundi	BDI
1	Africa	45	Comoros	COM
1	Africa	62	Ethiopia PDR	ETH
1	Africa	72	Djibouti	DJI
1	Africa	114	Kenya	KEN
1	Africa	129	Madagascar	MDG
1	Africa	130	Malawi	MWI
1	Africa	137	Mauritius	MUS
1	Africa	144	Mozambique	MOZ
1	Africa	178	Eritrea	ERI
1	Africa	181	Zimbabwe	ZWE
1	Africa	182	Réunion	REU
1	Africa	184	Rwanda	RWA
1	Africa	196	Seychelles	SYC
1	Africa	201	Somalia	SOM
1	Africa	215	United Republic of Tanzania	TZA
1	Africa	226	Uganda	UGA
1	Africa	238	Ethiopia	ETH
1	Africa	251	Zambia	ZMB
1	Africa	270	Mayotte	MYT
1	Africa	277	South Sudan	SSD
1	Africa	7	Angola	AGO
1	Africa	32	Cameroon	CMR
1	Africa	37	Central African Republic	CAF
1	Africa	39	Chad	TCD
1	Africa	46	Congo	COG
1	Africa	61	Equatorial Guinea	GNQ
1	Africa	74	Gabon	GAB
1	Africa	193	Sao Tome and Principe	STP
1	Africa	250	Democratic Republic of the Congo	COD
1	Africa	20	Botswana	BWA
1	Africa	122	Lesotho	LSO
1	Africa	147	Namibia	NAM
1	Africa	202	South Africa	ZAF
1	Africa	209	Swaziland	SWZ
1	Africa	35	Cabo Verde	CPV
1	Africa	53	Benin	BEN
1	Africa	75	Gambia	GMB
1	Africa	81	Ghana	GHA
1	Africa	90	Guinea	GIN
1	Africa	107	Côte d'Ivoire	CIV
1	Africa	123	Liberia	LBR
1	Africa	133	Mali	MLI

Code Color	Continent	Country code in FAO	Country	ISO code
1	Africa	136	Mauritania	MRT
1	Africa	158	Niger	NER
1	Africa	159	Nigeria	NGA
1	Africa	175	Guinea-Bissau	GNB
1	Africa	187	Saint Helena, Ascension and Tristan da Cunha	SHN
1	Africa	195	Senegal	SEN
1	Africa	197	Sierra Leone	SLE
1	Africa	217	Togo	TGO
1	Africa	233	Burkina Faso	BFA
1	Africa	4	Algeria	DZA
1	Africa	59	Egypt	EGY
1	Africa	124	Libya	LBY
1	Africa	143	Morocco	MAR
1	Africa	205	Western Sahara	ESH
1	Africa	206	Sudan (former)	SDN
1	Africa	222	Tunisia	TUN
1	Africa	276	Sudan	SDN
6	Europe	27	Bulgaria	BGR
6	Europe	51	Czechoslovakia	CSR
6	Europe	57	Belarus	BLR
6	Europe	97	Hungary	HUN
6	Europe	146	Republic of Moldova	MDA
6	Europe	167	Czech Republic	CZE
6	Europe	173	Poland	POL
6	Europe	183	Romania	ROU
6	Europe	185	Russian Federation	RUS
6	Europe	199	Slovakia	SVK
6	Europe	228	USSR	USS
6	Europe	230	Ukraine	UKR
6	Europe	54	Denmark	DNK
6	Europe	63	Estonia	EST
6	Europe	64	Faroe Islands	FRO
6	Europe	67	Finland	FIN
6	Europe	99	Iceland	ISL
6	Europe	104	Ireland	IRL
6	Europe	119	Latvia	LVA
6	Europe	126	Lithuania	LTU
6	Europe	162	Norway	NOR
6	Europe	210	Sweden	SWE
6	Europe	229	United Kingdom	GBR
6	Europe	259	Channel Islands	CHA
6	Europe	260	Svalbard and Jan Mayen Islands	SJM
6	Europe	264	Isle of Man	IMN
6	Europe	3	Albania	ALB
6	Europe	150	Netherlands	NLD
6	Europe	80	Bosnia and Herzegovina	BIH
6	Europe	82	Gibraltar	GIB
6	Europe	84	Greece	GRC
6	Europe	94	Holy See	VAT
6	Europe	98	Croatia	HRV
6	Europe	106	Italy	ITA
6	Europe	134	Malta	MLT
6	Europe	154	The former Yugoslav Republic of Macedonia	MKD
6	Europe	79	Germany	DEU
6	Europe	248	Yugoslav SFR	YSF
6	Europe	174	Portugal	PRT

Code Color	Continent	Country code in FAO	Country	ISO code
6	Europe	186	Serbia and Montenegro	SCG
6	Europe	192	San Marino	SMR
6	Europe	198	Slovenia	SVN
6	Europe	203	Spain	ESP
6	Europe	248	Yugoslav SFR	YSFR
6	Europe	272	Serbia	SRB
6	Europe	273	Montenegro	MNE
6	Europe	11	Austria	AUT
6	Europe	256	Luxembourg	LUX
6	Europe	15	Belgium-Luxembourg	BLX
6	Europe	140	Monaco	MCO
6	Europe	68	France	FRA
6	Europe	6	Andorra	AND
6	Europe	125	Liechtenstein	LIE
6	Europe	211	Switzerland	CHE
6	Europe	255	Belgium	BEL
6	Europe	256	Luxembourg	LUX
6	Europe	31	Bouvet Island	BVT
7	Americas	17	Bermuda	BMU
7	Americas	33	Canada	CAN
7	Americas	85	Greenland	GRL
7	Americas	190	Saint Pierre and Miquelon	SPM
7	Americas	231	United States of America	USA
7	Americas	111	Johnston Island	UMI
7	Americas	23	Belize	BLZ
7	Americas	48	Costa Rica	CRI
7	Americas	60	El Salvador	SLV
7	Americas	89	Guatemala	GTM
7	Americas	95	Honduras	HND
7	Americas	138	Mexico	MEX
7	Americas	157	Nicaragua	NIC
7	Americas	166	Panama	PAN
7	Americas	8	Antigua and Barbuda	ATG
7	Americas	12	Bahamas	BHS
7	Americas	14	Barbados	BRB
7	Americas	22	Aruba	ABW
7	Americas	36	Cayman Islands	CYM
7	Americas	49	Cuba	CUB
7	Americas	55	Dominica	DMA
7	Americas	56	Dominican Republic	DOM
7	Americas	86	Grenada	GRD
7	Americas	87	Guadeloupe	GLP
7	Americas	93	Haiti	HTI
7	Americas	109	Jamaica	JAM
7	Americas	135	Martinique	MTQ
7	Americas	142	Montserrat	MSR
7	Americas	151	Netherlands Antilles	ANT
7	Americas	177	Puerto Rico	PRI
7	Americas	188	Saint Kitts and Nevis	KNA
7	Americas	189	Saint Lucia	LCA
7	Americas	191	Saint Vincent and the Grenadines	VCT
7	Americas	220	Trinidad and Tobago	TTO
7	Americas	224	Turks and Caicos Islands	TCA
7	Americas	239	British Virgin Islands	VGB
7	Americas	240	United States Virgin Islands	USA
7	Americas	258	Anguilla	AIA
7	Americas	279	Curaçao	CUW

Code Color	Continent	Country code in FAO	Country	ISO code
7	Americas	280	Sint Maarten (Dutch Part)	SXM
7	Americas	9	Argentina	ARG
7	Americas	19	Bolivia (Plurinational State of)	BOL
7	Americas	21	Brazil	BRA
7	Americas	40	Chile	CHL
7	Americas	44	Colombia	COL
7	Americas	58	Ecuador	ECU
7	Americas	65	Falkland Islands (Malvinas)	FLK
7	Americas	69	French Guiana	GUF
7	Americas	91	Guyana	GUY
7	Americas	169	Paraguay	PRY
7	Americas	170	Peru	PER
7	Americas	207	Suriname	SUR
7	Americas	234	Uruguay	URY
7	Americas	236	Venezuela (Bolivarian Republic of)	VEN
10	Asia	108	Kazakhstan	KAZ
10	Asia	113	Kyrgyzstan	KGZ
10	Asia	208	Tajikistan	TJK
10	Asia	213	Turkmenistan	TKM
10	Asia	235	Uzbekistan	UZB
10	Asia	152	Neutral Zone	NTZ
10	Asia	41	China, mainland	CHN
10	Asia	96	China, Hong Kong SAR	CHN
10	Asia	214	China, Taiwan Province of	TWN
10	Asia	351	China	CHN
10	Asia	116	Democratic People’s Republic of Korea	PRK
10	Asia	117	Republic of Korea	KOR
10	Asia	128	China, Macao SAR	MAC
10	Asia	141	Mongolia	MNG
10	Asia	110	Japan	JPN
10	Asia	357	China (exc. Hong Kong & Macao)	CHN
10	Asia	2	Afghanistan	AFG
10	Asia	16	Bangladesh	BGD
10	Asia	18	Bhutan	BTN
10	Asia	38	Sri Lanka	LKA
10	Asia	100	India	IND
10	Asia	102	Iran (Islamic Republic of)	IRN
10	Asia	132	Maldives	MDV
10	Asia	149	Nepal	NPL
10	Asia	165	Pakistan	PAK
10	Asia	26	Brunei Darussalam	BRN
10	Asia	28	Myanmar	MMR
10	Asia	101	Indonesia	IDN
10	Asia	115	Cambodia	KHM
10	Asia	120	Lao People’s Democratic Republic	LAO
10	Asia	131	Malaysia	MYS
10	Asia	171	Philippines	PHL
10	Asia	176	Timor-Leste	TLS
10	Asia	200	Singapore	SGP
10	Asia	216	Thailand	THA
10	Asia	237	Viet Nam	VNM
10	Asia	1	Armenia	ARM
10	Asia	13	Bahrain	BHR
10	Asia	50	Cyprus	CYP
10	Asia	52	Azerbaijan	AZE
10	Asia	73	Georgia	GEO
10	Asia	103	Iraq	IRQ
10	Asia	105	Israel	ISR
10	Asia	112	Jordan	JOR

Code Color	Continent	Country code in FAO	Country	ISO code
10	Asia	118	Kuwait	KWT
10	Asia	121	Lebanon	LBN
10	Asia	179	Qatar	QAT
10	Asia	194	Saudi Arabia	SAU
10	Asia	212	Syrian Arab Republic	SYR
10	Asia	221	Oman	OMN
10	Asia	223	Turkey	TUR
10	Asia	225	United Arab Emirates	ARE
10	Asia	249	Yemen	YEM
10	Asia	299	Occupied Palestinian Territory	PSE
18	Oceania	164	Pacific Islands Trust Territory	TTP
18	Oceania	5	American Samoa	ASM
18	Oceania	92	Heard and McDonald Islands	HMD
18	Oceania	10	Australia	AUS
18	Oceania	25	Solomon Islands	SLB
18	Oceania	42	Christmas Island	CXR
18	Oceania	43	Cocos (Keeling) Islands	CCK
18	Oceania	47	Cook Islands	COK
18	Oceania	66	Fiji	FJI
18	Oceania	70	French Polynesia	PYF
18	Oceania	83	Kiribati	KIR
18	Oceania	88	Guam	GUM
18	Oceania	127	Marshall Islands	MHL
18	Oceania	145	Micronesia (Federated States of)	FSM
18	Oceania	148	Nauru	NRU
18	Oceania	153	New Caledonia	NCL
18	Oceania	155	Vanuatu	VUT
18	Oceania	156	New Zealand	NZL
18	Oceania	160	Niue	NIU
18	Oceania	161	Norfolk Island	NFK
18	Oceania	163	Northern Mariana Islands	MNP
18	Oceania	164	Pacific Islands Trust Territory	TTPI
18	Oceania	168	Papua New Guinea	PNG
18	Oceania	172	Pitcairn Islands	PCN
18	Oceania	180	Palau	PLW
18	Oceania	218	Tokelau	TKL
18	Oceania	219	Tonga	TON
18	Oceania	227	Tuvalu	TUV
18	Oceania	242	Wake Island	UMI

Code Color	Continent	Country code in FAO	Country	ISO code
18	Oceania	243	Wallis and Futuna Islands	WLF
18	Oceania	244	Samoa	WSM
18	Oceania	34	Canton and Edenbury Islands	CTE
18	Oceania	25	Solomon Islands	SLB
18	Oceania	66	Fiji	FJI
18	Oceania	153	New Caledonia	NCL
18	Oceania	155	Vanuatu	VUT
18	Oceania	168	Papua New Guinea	PNG
18	Oceania	83	Kiribati	KIR
18	Oceania	88	Guam	GUM
18	Oceania	127	Marshall Islands	MHL
18	Oceania	145	Micronesia (Federated States of)	FSM
18	Oceania	148	Nauru	NRU
18	Oceania	163	Northern Mariana Islands	MNP
18	Oceania	164	Pacific Islands Trust Territory	TTPI
18	Oceania	180	Palau	PLW
18	Oceania	5	American Samoa	ASM
18	Oceania	47	Cook Islands	COK
18	Oceania	70	French Polynesia	PYF
18	Oceania	160	Niue	NIU
18	Oceania	172	Pitcairn Islands	PCN
18	Oceania	218	Tokelau	TKL
18	Oceania	219	Tonga	TON
18	Oceania	227	Tuvalu	TUV
18	Oceania	242	Wake Island	UMI
18	Oceania	243	Wallis and Futuna Islands	WLF
18	Oceania	244	Samoa	WSM
23	Antarctic Region	30	Antarctica	ATA
23	Antarctic Region	71	French Southern and Antarctic Territories	ATF
19	Unspecified	252	Unspecified	UNS

Table B. List of countries per continent and associated codes in FAOstat.

C Identification of the countries that vary in the network over the study period

Almost all countries have been nodes of the network since the beginning of the study period with a few exceptions. For example, in 1992, the USSR broke up and new Eastern European countries joined the network as distinct nodes. In addition, some French oversea territories disappeared from the network because they are counted with France from 2000 onwards. In the table C, we report the nodes that appeared or disappeared from one year to the other.

	**Nodes removed**		**New nodes**	
	**ISO code**	**Country**	**ISO code**	**Country**
1987	LIE	Liechtenstein	IOT	British Indian Ocean Territory
			TKL	Tokelau
1988	IOT	British Indian Ocean Territory	YEM	Yemen
			VGB	British Virgin Islands
			BTN	Bhutan
1989			LIE	Liechtenstein
			CCK	Cocos (Keeling) Islands
			IOT	British Indian Ocean Territory
			MCO	Monaco
1990	LIE	Liechtenstein	TLS	Timor-Leste
	TKL	Tokelau	PCN	Pitcairn Islands
	MCO	Monaco		
1991	IOT	British Indian Ocean Territory	UMI	Wake Island
	TTP	Tokelau	MNP	Northern Mariana Islands
	TLS	Timor-Leste	MHL	Marshall Islands
			FSM	Micronesia (Federated States of)
			PLW	Palau
1992	UMI	Wake Island	IOT	British Indian Ocean Territory
	PCN	Pitcairn Islands	SVN	Slovenia
	USS	USSR	HRV	Croatia
	YSF	Yugoslav SFR	LIE	Liechtenstein
			KAZ	Kazakhstan
			SCG	Serbia and Montenegro
			TKM	Turkmenistan
			GEO	Georgia
			BLR	Belarus
			TJK	Tajikistan
			LVA	Latvia
			MKD	The former Yugoslav Republic of Macedonia
			MDA	Republic of Moldova
			TLS	Timor-Leste
			LTU	Lithuania
			NTZ	Neutral Zone
			RUS	Russian Federation
			AZE	Azerbaijan
			UKR	Ukraine
			BIH	Bosnia and Herzegovina
			UZB	Uzbekistan
			EST	Estonia
			KGZ	Kyrgyzstan
			ARM	Armenia

	Nodes removed		New nodes	
	**ISO code**	**Country**	**ISO code**	**Country**
1993	CSR	Czechoslovakia	UMI	Wake Island
	NTZ	Neutral Zone	CZE	Czech Republic
			PCN	Pitcairn Islands
			AIA	Anguilla
			SVK	Slovakia
			MCO	Monaco
1994	UMI	Wake Island	TKL	Tokelau
			ATF	French Southern and Antarctic Territories
			ESH	Western Sahara
			SJM	Svalbard and Jan Mayen Islands
			ERI	American Samoa
1995	IOT	British Indian Ocean Territory	SMR	San Marino
	ESH	Western Sahara		
	TLS	Timor-Leste		
1996	SJM	Svalbard and Jan Mayen Islands	IOT	British Indian Ocean Territory
	AIA	Anguilla	ESH	Western Sahara
	MCO	Monaco		
1997	IOT	British Indian Ocean Territory	MCO	Monaco
1998	TKL	Tokelau	IOT	British Indian Ocean Territory
	PCN	Pitcairn Islands		
	MCO	Monaco		
1999			UMI	Wake Island
			PCN	Pitcairn Islands
			TKL	Tokelau
			TLS	Timor-Leste
			MCO	Monaco
2000	LIE	Liechtenstein	PSE	Occupied Palestinian Territory
	CCK	Cocos (Keeling) Islands	BEL	Belgium
	IOT	British Indian Ocean Territory	LUX	Luxembourg
	PRI	Puerto Rico		
	VAT	Holy See		
	UMI	Wake Island		
	SMR	San Marino		
	GIB	Gibraltar		
	CXR	Christmas Island		
	GUF	French Guiana		
	PCN	Pitcairn Islands		
	MNP	Northern Mariana Islands		
	MTQ	Martinique		
	BLX	Belgium Luxembourg		
	NFK	Norfolk Island		
	AND	Northern Mariana Islands		
	ATF	French Southern and Antarctic Territories		
	REU	Réunion		
	GLP	Guadeloupe		
	ESH	Western Sahara		
	TLS	Timor-Leste		
	MCO	Monaco		
2001	TKL	Tokelau	MYT	Mayotte
			TLS	Timor-Leste
2002				
2003			GIB	Gibraltar
			BVT	Bouvet Island
			LIE	Liechtenstein
			PCN	Pitcairn Islands
			HMD	Heard and McDonald Islands
			AIA	Anguilla

	Nodes removed		New nodes	
	**ISO code**	**Country**	**ISO code**	**Country**
2004	GIB<br>BVT<br>LIE<br>PCN<br>HMD<br>AIA	Gibraltar<br>Bouvet Island<br>Liechtenstein<br>Pitcairn Islands<br>Heard and McDonald Islands<br>Anguilla	ATF	French Southern and Antarctic Territories
2005	ATF	French Southern and Antarctic Territories		
2006	SCG	Serbia and Montenegro	MNE<br>MNP<br>ATF<br>SRB	Montenegro<br>Northern Mariana Islands<br>French Southern and Antarctic Territories<br>Serbia
2007	MNP<br>ATF	Northern Mariana Islands<br>French Southern and Antarctic Territories		
2008			AIA	Anguilla
2009	AIA	Anguilla		
2010			AND	Northern Mariana Islands
2011	AND<br>ANT	Northern Mariana Islands<br>Netherlands Antilles		
2012	SDN	Sudan (former)		
2013				

Table C. Identification of the countries that appear or disappear in the network over the study period.

D Consistency of data

In the FAOstat database, the detailed trade matrix provides trades from a country (i) to another country (j) for each year between 1986 and 2013 included. Countries report the quantities exported per country of destination as well as the quantities imported per country of origin. In principle, all trade must be reported twice: once as a quantity imported by the destination country and once as a quantity exported by the country of origin with the same value. Comparing both matrices, we notice two types of discrepancy: on the one hand the mass of the trade is not equal in tons and in the other hand some trades are only reported once (by the exporting country or the importing country). Those discrepancies are justified by the FAO[1]: certain commodities can be classed differently between the country of destination and the country of origin, some products are lost during the transportation, time lag (export reported in December, and the import is reported during the following year), data confidentiality by one of the partners. Choosing between one of the two matrices corresponds to making a strong hypothesis, and there is no certain way of knowing which country has reported the right value to the FAO. To mitigate the discrepancies, we choose to keep the average of the two values for our study.

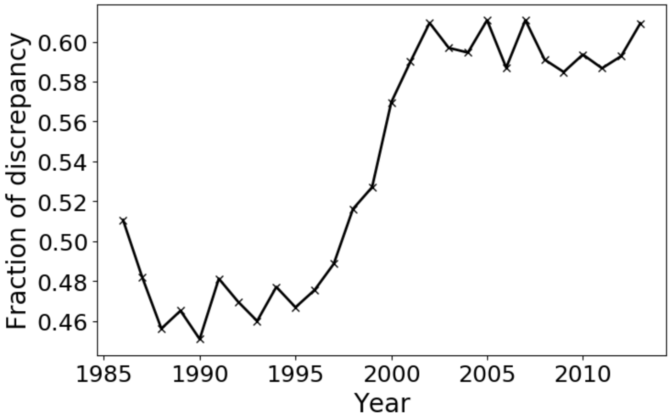

Figure A. Fraction of number of discrepancies to total number of trades. An error occurs when the trade is reported only once or when there is a discrepancy in the traded value reported by both partner countries. The two types of errors are aggregated in this figure. Although the fraction of errors is large, the differences in mass are small, see Fig. C.

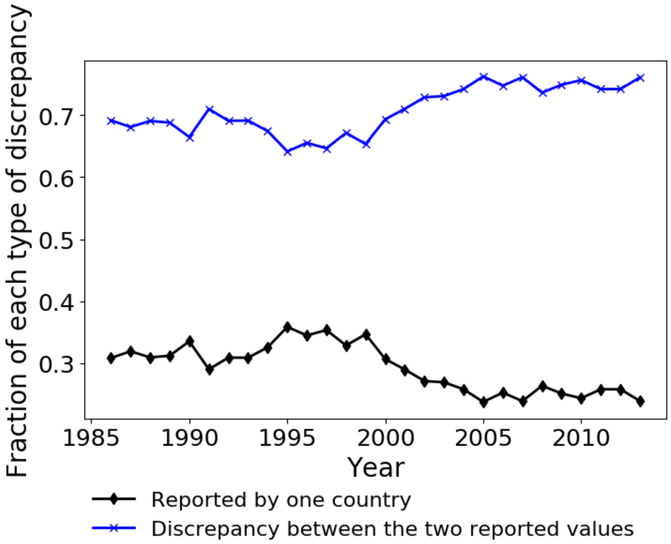

Figure B. Fraction of the type of discrepancy as a function of time. The majority of discrepancies concern different mass value reported between the exporting and the importing country (blue line). The other type of error occurs when the trade is reported only once either by the exporting country or the importing country (black line).

The share of trades on which there is no full agreement between the exporting country and the importing country increased from 51% in 1986 to 61% in 2000 (Fig. A). The ratio of the two types of discrepancy are reported in Fig. B. The case where the trade is reported only once represent a minority of 30% of the total number of discrepancies in 1986 and decreases to 24% in

2013.

The discrepancies are significant since they affect more than half of the total trades. However, the discrepancies are lower in mass than in total number of discrepancies. We calculate the variability of the mass per year:

$$\Delta \hat{W}_y = \frac{\sum |w_1 - w_2|}{\sum \bar{w}} \quad (1)$$

where w_1 and w_2 are respectively the trade value reported by the node 1 and the trade value reported the node 2, w_i is equal to zero if the trade is not reported, $\bar{w}$ is the average between w_1 and w_2, and $\hat{W}_y$ is the weighted proportion of discrepancy of the year y.

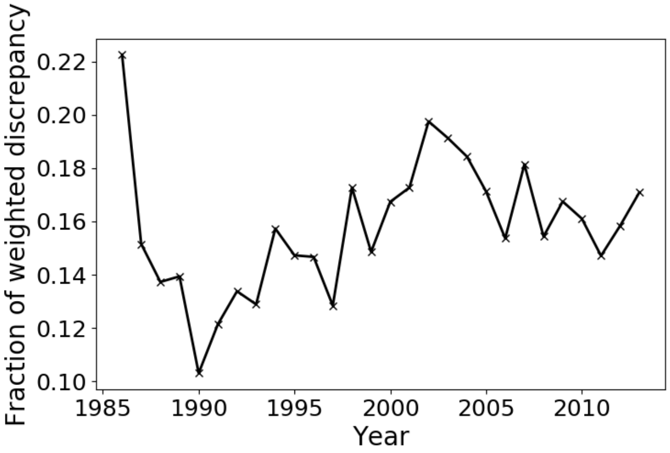

Figure C. Weighted fraction of discrepancies in total traded mass. A discrepancy occurs when a trade is reported only once or when there is a divergence in the traded value reported by both partner countries. The two types of errors are aggregated in this figure. The weighted errors fluctuate around 16%.

The weighed discrepancy in 2013 is 17% (Fig. D). During the study period, the weighed discrepancy varies between 10% in 1990 and 22% in 1986. The weighted discrepancy is significant but acceptable.

The distribution of the mass discrepancies shows that only a few exchanges represent great mass differences (Fig. E). The range of the mass discrepancy is between 1 ton and 4 million tons on the study period.

We define the relative discrepancy for a trade as:

$$x = |\frac{\bar{w} - min(w_1, w_2)}{\bar{w}}| \quad (2)$$

where w_1 and w_2 are respectively the trade reported by node 1 and the trade reported by node 2; w_i is equal to zero if the trade is not reported. $\bar{w}$ is the average between w_1 and w_2.

We weight the relative error, x, with the trading capacity of the node:

$$x_w = x \times \frac{w}{w_{max}} \quad (3)$$

where w_{max} is the maximum traded mass of the node and w is the mass value of the trade.

Most of the discrepancies correspond to a small share of the trading capacity of the country. The distribution of the relative discrepancies weighted by the largest trade of the country follows a power law function. This result means that only a minority of countries have large mass discrepancies in comparison to their total traded mass such as Serbia (95%), Switzerland (84%), Moldova (56%). In contrast many countries have small discrepancies.

E Subnetwork description.

The **transient subnetwork**, comprising trades of short duration *i.e* of 1 or 2 years, gathers about two thirds of all trades but only accounts for 1.7% of the cumulative traded mass over the study period. Their share in total traded mass dropped from 2.1% to 0.8% (Fig. F.B). Nevertheless, the number of links has doubled from 597 in 1986 to 1210 in 2013 (Fig. F.A).

The trades of duration comprised between 3 and 27 consecutive years define the **intermediate subnetwork**. The stability of these trades increases over time. The intermediate subnetwork grew the most significantly during the study period, from

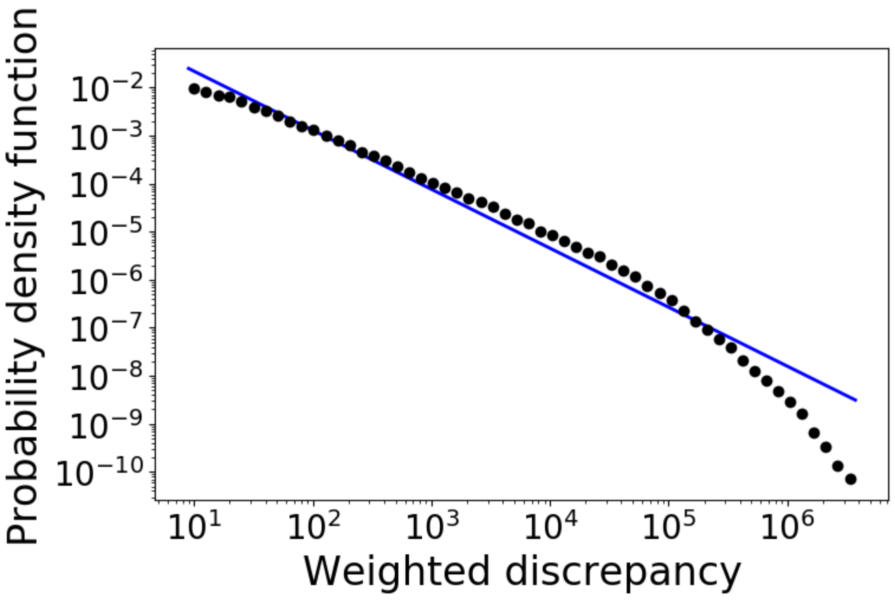

Figure D. **Distribution of weighted discrepancies in FAOstat data matrices (1986-2013).** The probability density function is fitted with a linear function in a log-log scale which means that the fitting function is a power law, x^{α}. The parameter $\alpha = -1.22$.

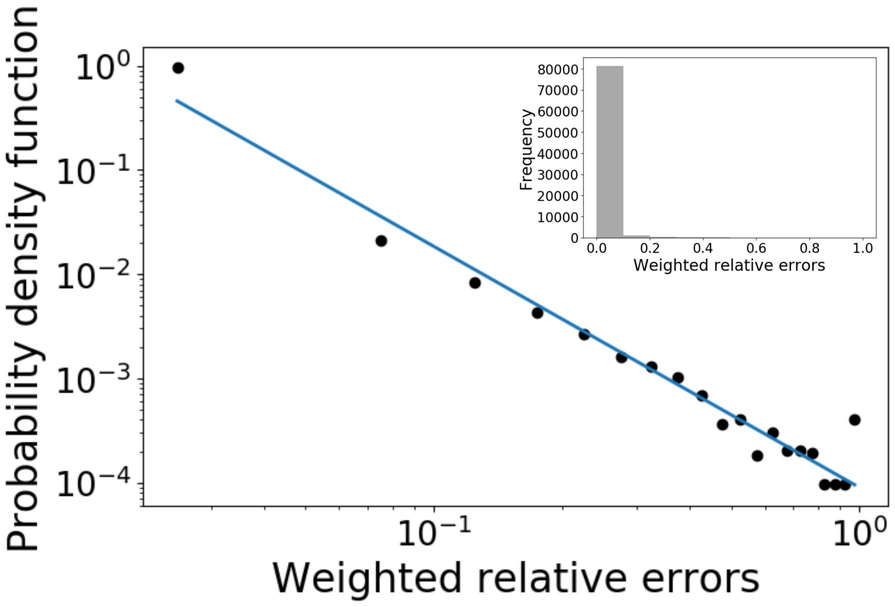

Figure E. **Distribution of weighted relative discrepancies in FAOstat data matrices (1986-2013).** The probability density function is fitted with a linear function in a log-log scale which means that the fitting function is a power law, x^{α}. The parameter $\alpha = -2.31$ and $R^2 = 0.98$. The inset is the histogram of the distribution.

about 1047 links in 1986 to 5422 links in 2013 (Fig. F.A). The traded share mass increased from 15% of all cereal mass traded in 1986 to 42% in 2013 (Fig. F.B).

The third and last class contains uninterrupted trades throughout the study period (lasting 28 years or more). This class defines the **backbone subnetwork**. In 1986, the backbone subnetwork accounts for 83% of all traded mass, and in 2013 it accounts for 58%.

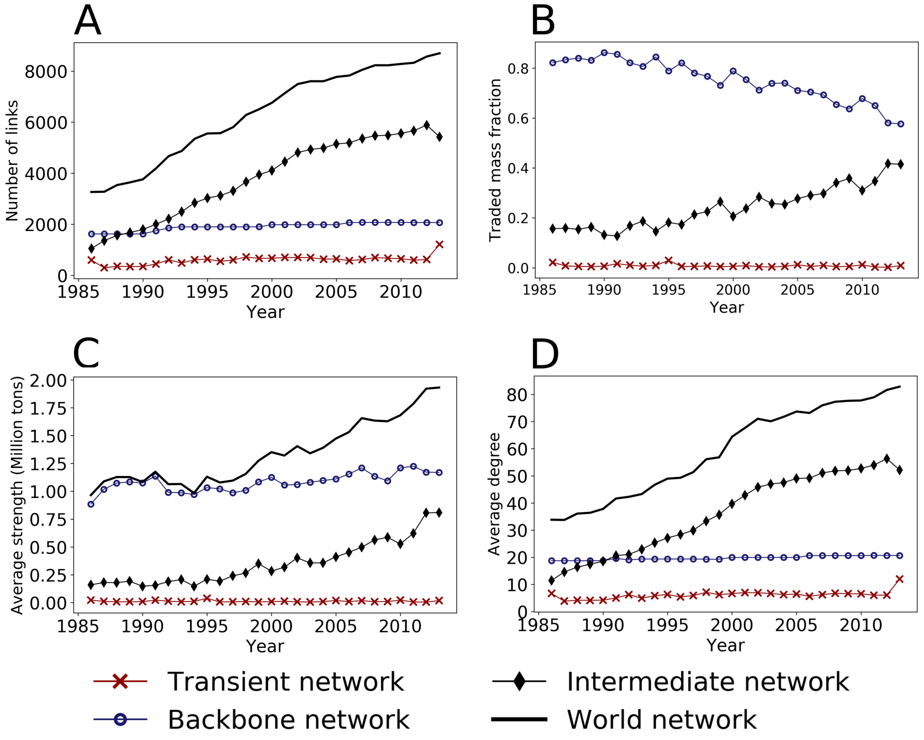

Figure F. **A. Number of links (number of trades) as a function of time for each subnetwork and for the total network.** The intermediate subnetwork is growing and the backbone and transient subnetworks are stable in the number of links. **B. Traded mass fraction for each subnetwork as function of time.** The intermediate subnetwork is growing in term of traded mass and the backbone is decreasing. **C. Average node strength as a function of time for each subnetwork and for the total network.** The node strength is defined as the total mass traded by a country (node). **D. Average node degree as a function of time for each subnetwork and for the total network.** The node degree is the number of trades for a country (node).
