# Supplementary material for: Time dynamics and invariant subnetwork structures in the world cereals trade network
Source: PLoS One. 2019 May 22;14(5):e0216318. doi: 10.1371/journal.pone.0216318 (PMC6530828; doi:10.1371/journal.pone.0216318)
Supplement: S2 Appendix — (PDF) [file pone.0216318.s002.pdf]

S2 Appendix. Connectivity of the world cereals trade network

A Structure Evolution of the global cereal trade network

The topology of the total network has changed from a scale free to an exponential degree distribution. The node degree distribution scaled with a power law fit in 1986 while fitted with an exponential distribution in 2013. The power law distribution reflects the scale-free property of the network. The degree distribution change is shown by the decrease of the loglikelihood test between the two fits G. We compute the loglikehood ratio from the powerlaw package[2] to identify the best fit. Trades that appeared after 1986 have not followed the rule of preferential attachment. As the number of nodes remains constant, new links connect nodes that are already present in the network. This transition highlights the emergence of new powerful nodes, mostly corresponding to developing countries with high agricultural potential previously under-exploited.

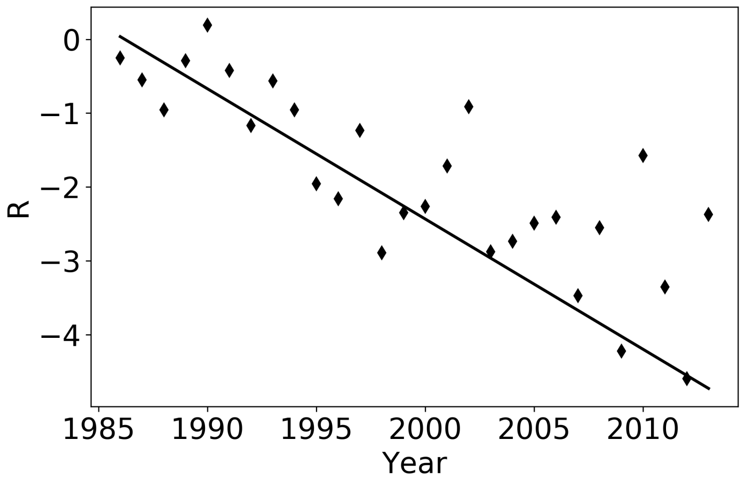

Figure G. Log-likehood between the power law distribution and the exponential distribution.

B Nodes strength vs nodes degree

The node strength is the sum of quantities that is traded by this node, it is the sum of import and the sum of exports. We distinguish the node in-strength that is only the sum of imports and the out-strength that is the sum of export. For the world cereals trade network, the node strength follows a power law as a function of the node degree (Fig. H.A). The relationship is also valid for the node out-strength (Fig. H.C), the node in-strength (Fig. H.D) of the total world network and also for all three subnetworks (Fig. I).

C Assortativity analysis of the three subnetworks

For the entire study period, the backbone subnetwork exhibits "disassortative" behaviour as the average degree of nearest neighbours of nodes decrease with the node degree (Fig.J.A). The intermediate subnetwork exhibits disassortative behaviour in 1986 and it becomes more assortative over time (Fig.J.B).

D Nodes degree in the backbone subnetwork and the intermediate subnetwork

In order to understand the emerging dynamics of the intermediate subnetwork, we compare its evolution of node connectivity with the evolution of the backbone subnetwork. The node degree between the two subnetworks relate through a logistic function (Figs. K). The node connectivity increases rapidly in the intermediate subnetwork and there are less and less nodes with lower connectivity than in the backbone subnetwork. Since 2000, only the five major hubs have higher connectivity in the backbone subnetwork: Belgium, France, the United States, the Netherlands and Italy.

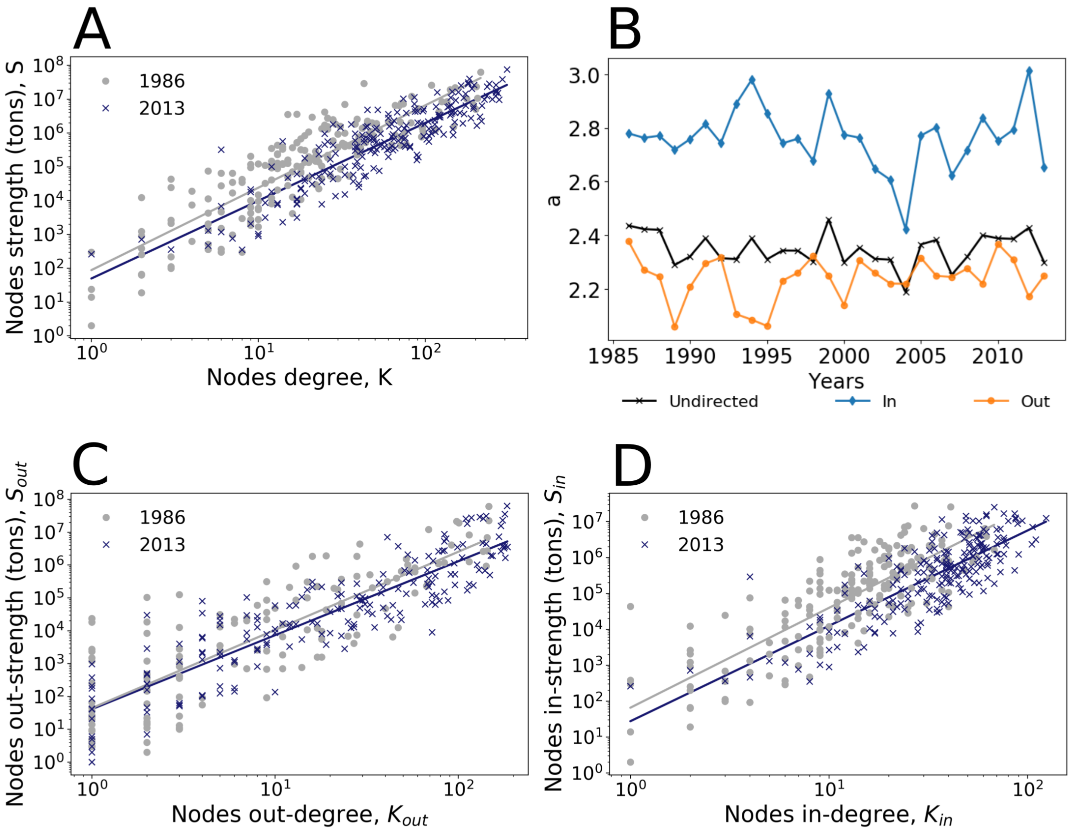

Figure H. **A. Node strength as function of node degree. B. Parameter of power law fitting as function of time.** Black line is the parameter of the power law fitting for the node strength as function of node degree (Fig. H.A), Blue line is the parameter of the power law fitting for the node In-strength as function of node In-degree (Fig. H.D) and Orange line is the parameter of the power law fitting for the node Out-strength as function of node Out-degree (Fig. H.C). **C. Node Out-strength as function of node Out-degree. D. Node In-strength as function of node In-degree.**

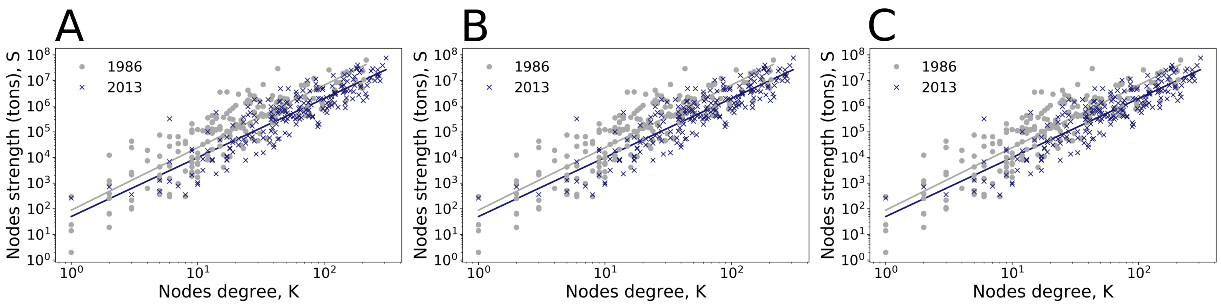

Figure I. A. Node strength as function of node degree of the backbone subnetwork. B. Node strength as function of node degree of the intermediate subnetwork. C. Node strength as a function of node degree of the transient subnetwork.

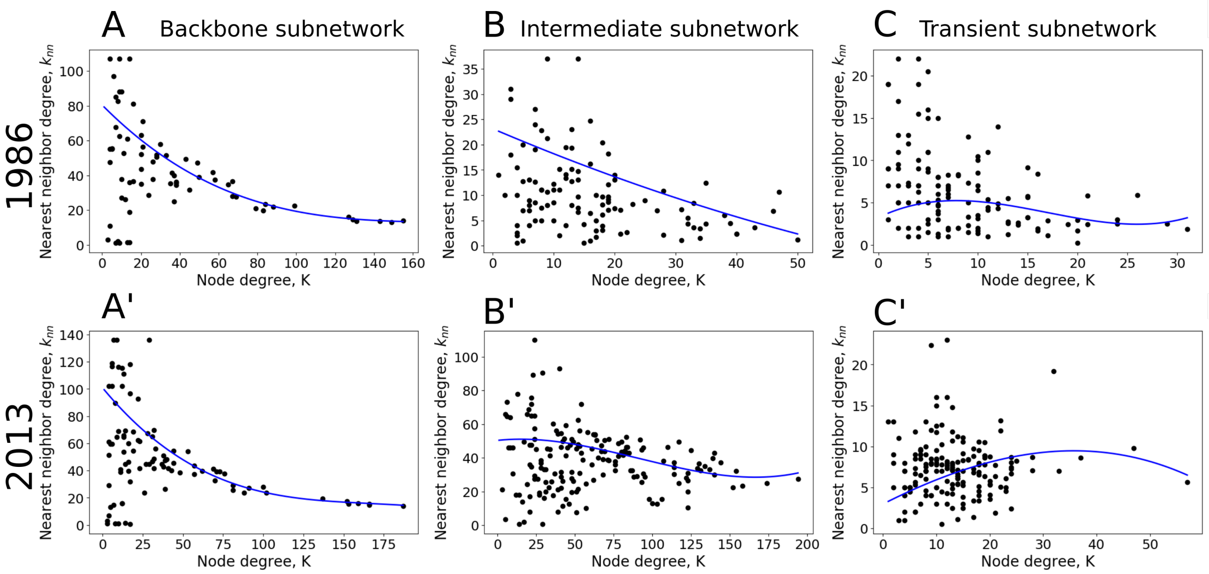

Figure J. **Degree of nearest neighbours to node as a function of the node degree.** Each black dot corresponds to node values with as x-axis the number of trades of the node (k), and as y-axis the average degree of the nearest neighbour to the node. The solid line shows the tendency fit with a polynomial function of three degrees. **A & A'.** Degree of nearest neighbours to node in the backbone subnetwork for 1986 and 2013. **B & B'.** Degree of nearest neighbours to node in the intermediate subnetwork for 1986 and 2013. **C & C'.** Degrees of nearest neighbours to node in the transient subnetwork for 1986 and 2013.

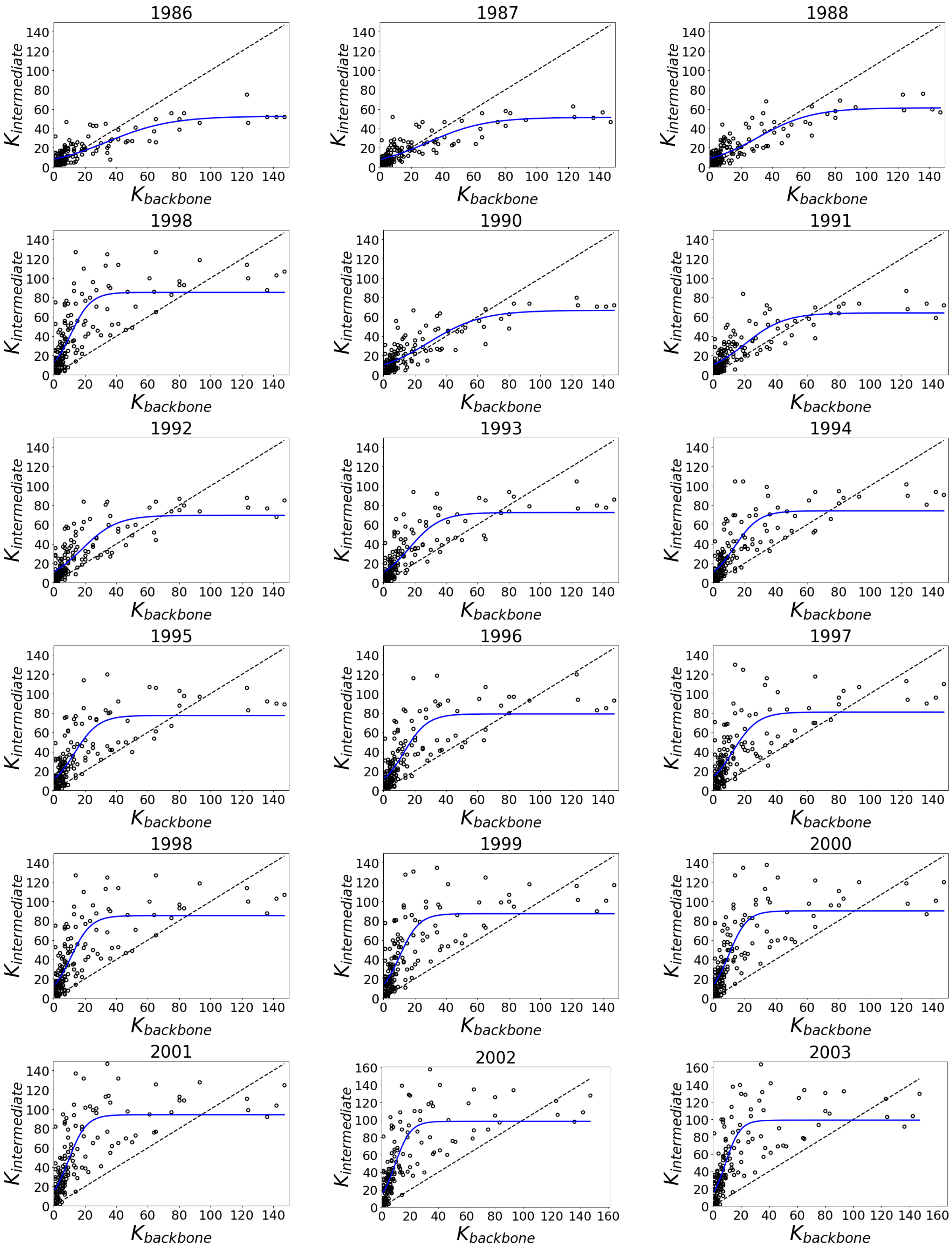
1986
1987
1988
1998
1990
1991
1992
1993
1994
1995
1996
1997
1998
1999
2000
2001
2002
2003
$K_{intermediate}$
$K_{backbone}$

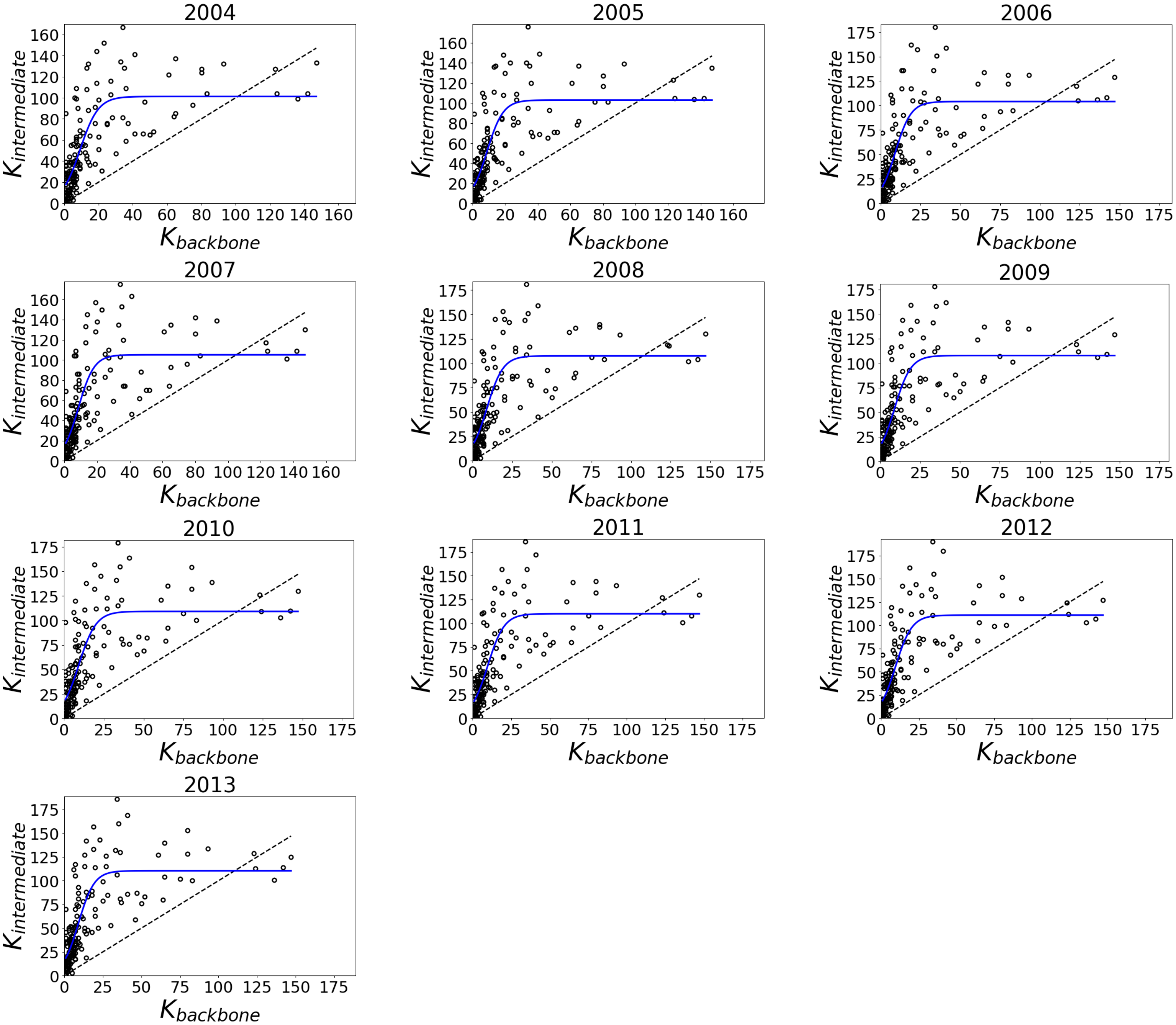

Figure K. Node degree in the intermediate subnetwork as a function of the node degree in the backbone subnetwork. The blue line is the fitting with a logistic equation: $K_i(K_b) = \frac{K_{max}}{1+ae^{-rK_b}}$. The dashed line is the line where nodes have the same number of links in the intermediate subnetwork than in the backbone subnetwork. Only five countries remain with more trades on the backbone subnetwork: Belgium, France, United States, Netherlands and Italy.
