# Supplementary material for: Time dynamics and invariant subnetwork structures in the world cereals trade network
Source: PLoS One. 2019 May 22;14(5):e0216318. doi: 10.1371/journal.pone.0216318 (PMC6530828; doi:10.1371/journal.pone.0216318)
Supplement: S3 Appendix — (PDF) [file pone.0216318.s003.pdf]

S3 Appendix. Network centrality measures

A Centrality measure of the backbone subnetwork

In the backbone subnetwork, the hubs remain the same countries over the study period (Table D). United States are the largest trader in terms of mass and Belgium is the highest connected (number of trades) and the most central country (betweeness centrality coefficient). We also observe a power law relationship between the node betweeness centrality coefficient and the node degree k (Fig. L). It indicates that connected nodes play a crucial roles into the backbone subnetwork.

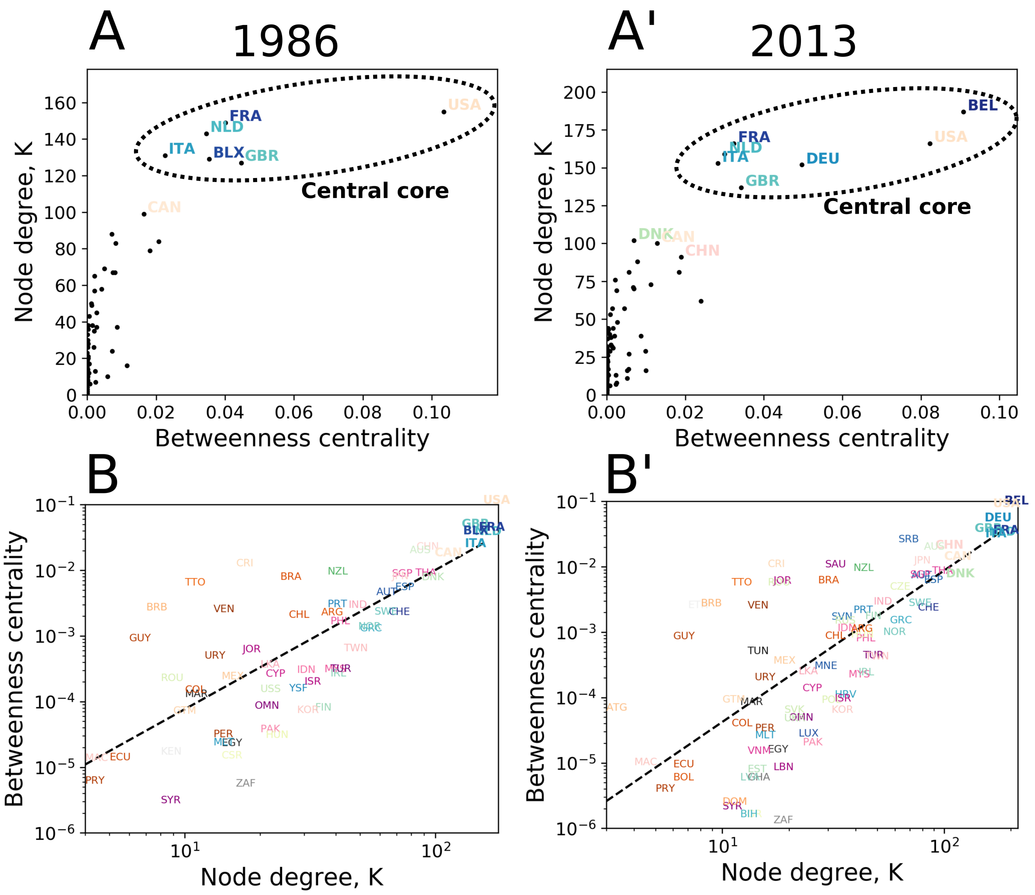

Figure L. Relationship between the node betweenness centrality and the node degree (k) for 1986 and 2013 in the backbone subnetwork. A, A'. Node degree (k) vs node betweeness centrality in 1986 and 2013. The core groups remain constant representing industrial European countries and United States. **B, B'. Nodes betweeness centrality vs node degree (k) in 1986 and 2013.** Color of country name corresponds to the color in the network visualization in Fig. 4 in the main text.

B Centrality measure of the intermediate subnetwork

In the intermediate subnetwork, the highest connected nodes change over the study period (Table E). During the period 1986-1995, the hubs are mainly historical industrial countries including Western European countries and the USA. Since the late 1990's, the intermediate subnetwork is being increasingly led by developing countries, mainly Asian and South American countries (Turkey, Malaysia, Pakistan, China Argentina and Brazil) as well as some Eastern European countries such as Poland, Ukraine and Russia. We also observe a power law relationship between the node betweeness centrality coefficient and the node degree k (Fig. M). However the highest connected nodes are not necessarily the highest central nodes (Tables F & G and Fig M.A').

Rank	Country	Number of trades	Strength (million tons)	Betweeness centrality	Closeness centrality
1	Belgium	187	12.3	0.091	0.295
2	United States	166	72.6	0.082	0.297
3	France	166	37.1	0.032	0.273
4	Netherlands	159	14.1	0.030	0.277
5	Italy	153	12.6	0.028	0.268
6	Germany	152	25.1	0.050	0.281
7	United Kingdom	137	8.4	0.034	0.279
8	Denmark	102	2.7	0.008	0.248
9	Canada	100	25.3	0.013	0.279

Table D. Measures of connectivity (degree of node) and centrality of the top 9th connected countries in the backbone subnetwork in 2013.

Rank	1986	1987	1988	1989
1	United Kingdom	United Kingdom	Denmark	Netherlands
2	Australia	France	Netherlands	United States of America
3	Denmark	Denmark	United Kingdom	United Kingdom
4	France	Sweden	Canada	Denmark
5	United States of America	Taiwan	Ireland	Canada
6	Sweden	Australia	United States of America	France
7	Taiwan	Netherlands	Belgium-Luxembourg	Italy
8	Canada	Belgium-Luxembourg	France	China
9	Saudi Arabia	Spain	Spain	Ireland
10	Netherlands	Republic of Korea	Australia	Australia

Rank	1990	1991	1992	1993
1	United Kingdom	United Kingdom	United Kingdom	United Kingdom
2	United States of America	Netherlands	Belgium-Luxembourg	Belgium-Luxembourg
3	Netherlands	Belgium-Luxembourg	United States of America	Pakistan
4	Denmark	Pakistan	Pakistan	Denmark
5	Canada	Denmark	Denmark	Turkey
6	Ireland	United States of America	Spain	United States of America
7	Italy	Turkey	Argentina	Switzerland
8	France	Canada	Turkey	China, mainland
9	China, mainland	Spain	China, mainland	Spain
10	Spain	Australia	Australia	Netherlands

Rank	1994	1995	1996	1997
1	United Kingdom	Belgium-Luxembourg	United Kingdom	South Africa
2	Turkey	Turkey	Pakistan	Pakistan
3	Belgium-Luxembourg	Pakistan	Belgium-Luxembourg	Spain
4	Pakistan	United Kingdom	Turkey	United Kingdom
5	Spain	Spain	Spain	Turkey
6	United States of America	Switzerland	Denmark	Argentina
7	China, mainland	Thailand	United States of America	United States of America
8	France	Denmark	Switzerland	Canada
9	Denmark	United States of America	Argentina	Belgium-Luxembourg
10	Switzerland	Canada	Thailand	Denmark

Rank	1998	1999	2000	2001
1	South Africa	Pakistan	Pakistan	Turkey
2	Spain	Turkey	Turkey	South Africa
3	Turkey	South Africa	South Africa	Pakistan
4	Canada	Spain	Spain	India
5	United States of America	United States of America	Canada	Canada
6	United Kingdom	Canada	United States of America	Spain
7	Pakistan	United Kingdom	India	United States of America
8	Argentina	India	United Kingdom	Argentina
9	India	Australia	China, mainland	China, mainland
10	France	France	Argentina	United Kingdom

Rank	2002	2003	2004	2005
1	Turkey	Turkey	Turkey	Turkey
2	United Arab Emirates	United Arab Emirates	Brazil	India
3	South Africa	South Africa	Pakistan	Pakistan
4	India	Pakistan	India	Brazil
5	Pakistan	India	South Africa	Argentina
6	Spain	Brazil	Argentina	Malaysia
7	Canada	Spain	Spain	Egypt
8	United States of America	Canada	Egypt	Spain
9	United Kingdom	United States of America	United States of America	United States of America
10	Argentina	Argentina	Canada	South Africa

Rank	2006	2007	2008	2009
1	Turkey	Turkey	Turkey	Turkey
2	India	United Arab Emirates	United Arab Emirates	India
3	Brazil	Brazil	India	Malaysia
4	Pakistan	India	Pakistan	Pakistan
5	Malaysia	Pakistan	Malaysia	Poland
6	Egypt	Malaysia	Brazil	South Africa
7	South Africa	South Africa	South Africa	Argentina
8	Argentina	Argentina	Argentina	Brazil
9	Spain	Spain	China, mainland	China, mainland
10	China, mainland	China, mainland	Poland	Thailand

Rank	2010	2011	2012	2013
1	Turkey	Turkey	Turkey	Turkey
2	Malaysia	India	India	India
3	India	Malaysia	Malaysia	Malaysia
4	Pakistan	Pakistan	Pakistan	Pakistan
5	China, mainland	China, mainland	China, mainland	China, mainland
6	Poland	Brazil	Poland	Poland
7	Brazil	Poland	Brazil	Russian Federation
8	Argentina	Spain	Ukraine	Spain
9	South Africa	Russian Federation	Spain	Ukraine
10	Spain	Argentina	Russian Federation	Brazil

Table E. Tables of the ten most important hubs in the intermediate subnetwork for the study period (1986-2013) sorted by number of trades. Colors are assigned by continent. Europe in blue, North and central America in orange, South America in red, Africa in grey, East Asia in purple, Asia in pink and Oceania in green.

Country	Degree of node	Betweenness centrality	Closeness centrality
Turkey	194	0.034	0.443
India	174	0.025	0.436
Malaysia	158	0.018	0.402
Pakistan	154	0.021	0.436
China	152	0.018	0.400
Poland	144	0.008	0.393
Russia	140	0.021	0.423
Spain	140	0.018	0.417
Ukraine	139	0.012	0.421
Brazil	137	0.008	0.408
South Africa	134	0.037	0.447
Argentina	131	0.008	0.395
Republic of Korea	130	0.017	0.414

Table F. Measures of connectivity (node degree) and centrality of the top 13 connected countries in the intermediate subnetwork in 2013 sorted by the node degree.

Country	Degree of node	Betweenness centrality	Closeness centrality
United States	123	0.047	0.457
Canada	122	0.0405	0.444
United Kingdom	123	0.0380	0.453
South Africa	134	0.0369	0.447
Turkey	194	0.0342	0.443
France	103	0.0310	0.451
Switzerland	123	0.0257	0.431
India	174	0.0248	0.436
Pakistan	154	0.0209	0.436
Russia	140	0.0206	0.423
China	152	0.0184	0.400
Malaysia	158	0.0180	0.402
Spain	140	0.0179	0.417
Republic of Korea	130	0.0173	0.414

Table G. Measures of connectivity (node degree) and centrality of the top 13 connected countries in the intermediate subnetwork in 2013 sorted by the betweenness centrality node.

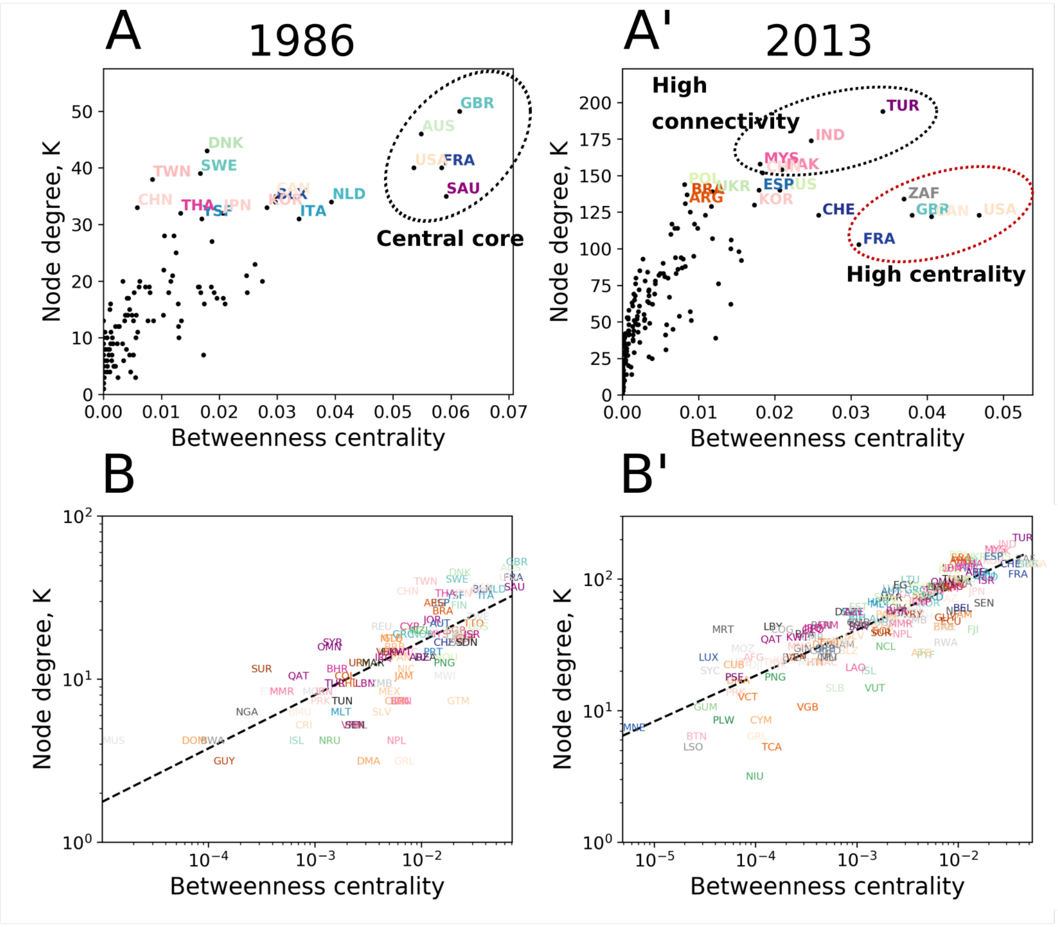

Figure M. **Relationship between the node betweenness centrality and the node degree (k) for 1986 and 2013 in the intermediate subnetwork. A, A'. Node degree (k) vs node betweeness centrality in 1986 and 2013.** In 1986, the core group is composed by United Kingdom, Australia, United States, France and Saudi Arabia. In 2013, the high connectivity countries are Turkey, India, Malaysia, Pakistan and China. The highest central countries are United States, Canada, United Kingdom and France. **B, B'. Node degree (k) vs node betweeness centrality in 1986 and 2013 on log-log axis.** Color of country name corresponds to the color in network visualization in Fig. 4 in the main text.
