# Supplementary material for: Time dynamics and invariant subnetwork structures in the world cereals trade network
Source: PLoS One. 2019 May 22;14(5):e0216318. doi: 10.1371/journal.pone.0216318 (PMC6530828; doi:10.1371/journal.pone.0216318)
Supplement: S4 Appendix — (PDF) [file pone.0216318.s004.pdf]

S4 Appendix. Characteristics of the cereals production for some countries

For the most connected countries in the network, we extract from the FAOstat the characteristic of their cereal production. For all figures below, the blue line is the amount of cereal harvested area, the red line is the yield (i.e. the production of cereals for a unit of area harvested) and the yellow line is total cereal production. We observe that for the majority of the countries and especially for the hubs in the intermediate subnetwork, such as Turkey, Malaysia, Poland, Russia, and Spain, the harvested area decreases during the study period but the production increases thanks to the growth of their yields (Figs. N10, N12, N15, N16 and N17). China and Pakistan clearly extend their area harvested as well as their yields and cereal productions (Figs. N14) & N13).

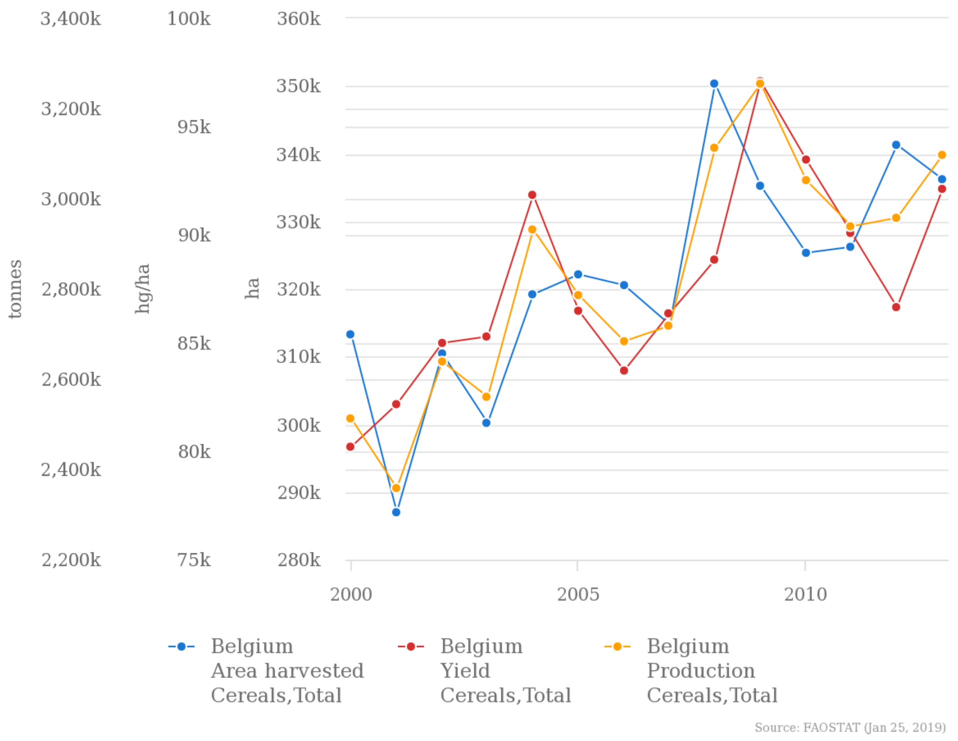

Figure N1. **Cereal production of Belgium.**

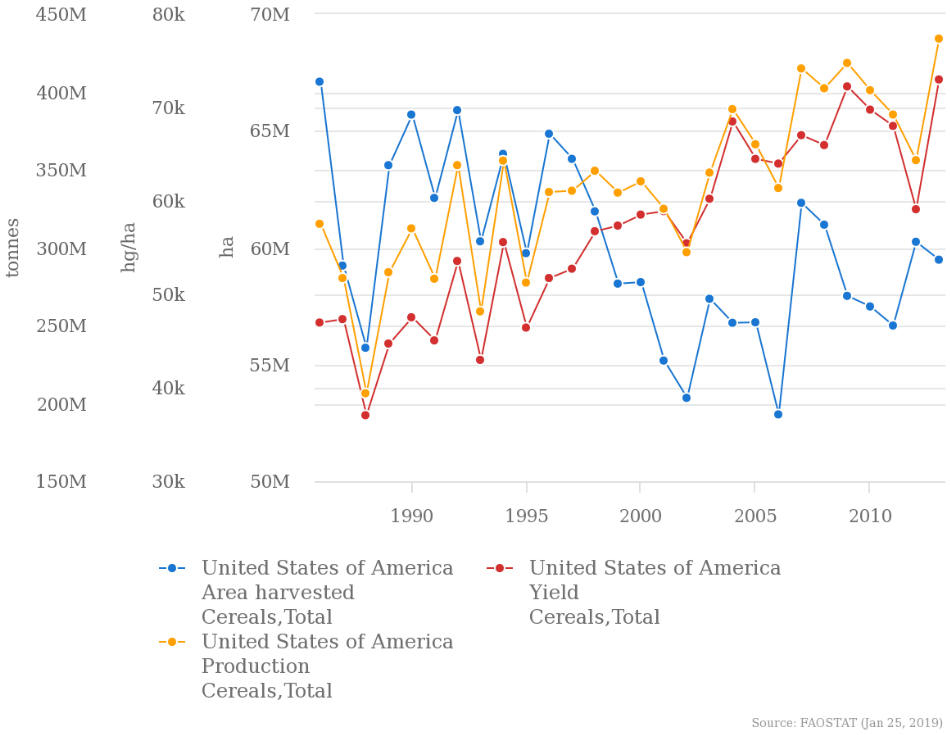

Figure N2. **Cereal production of United States.**

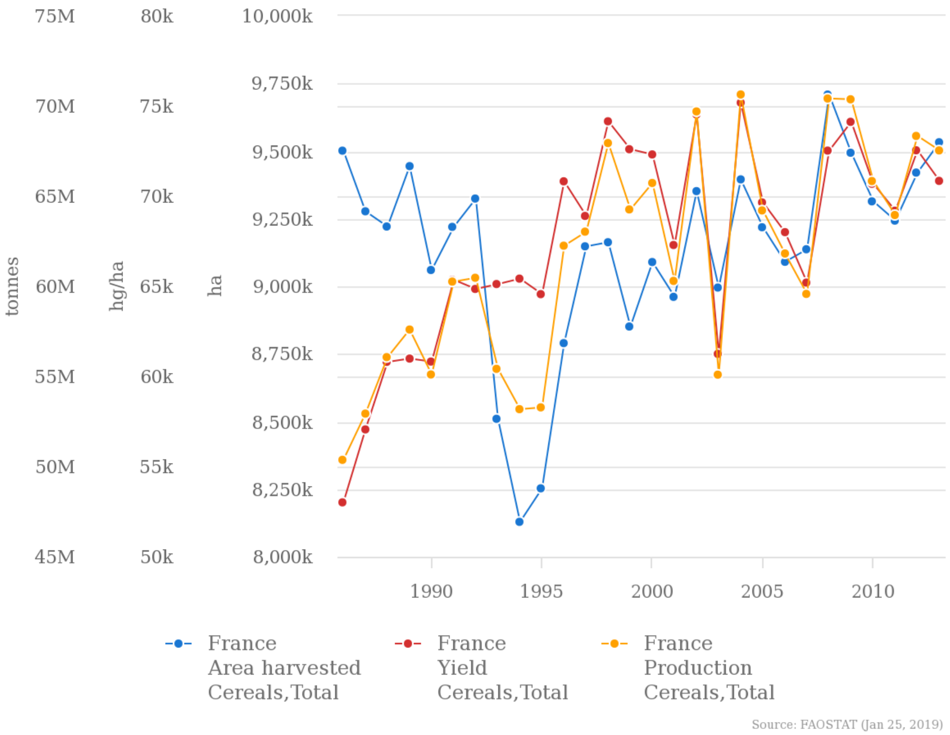

Figure N3. **Cereal production of France.**

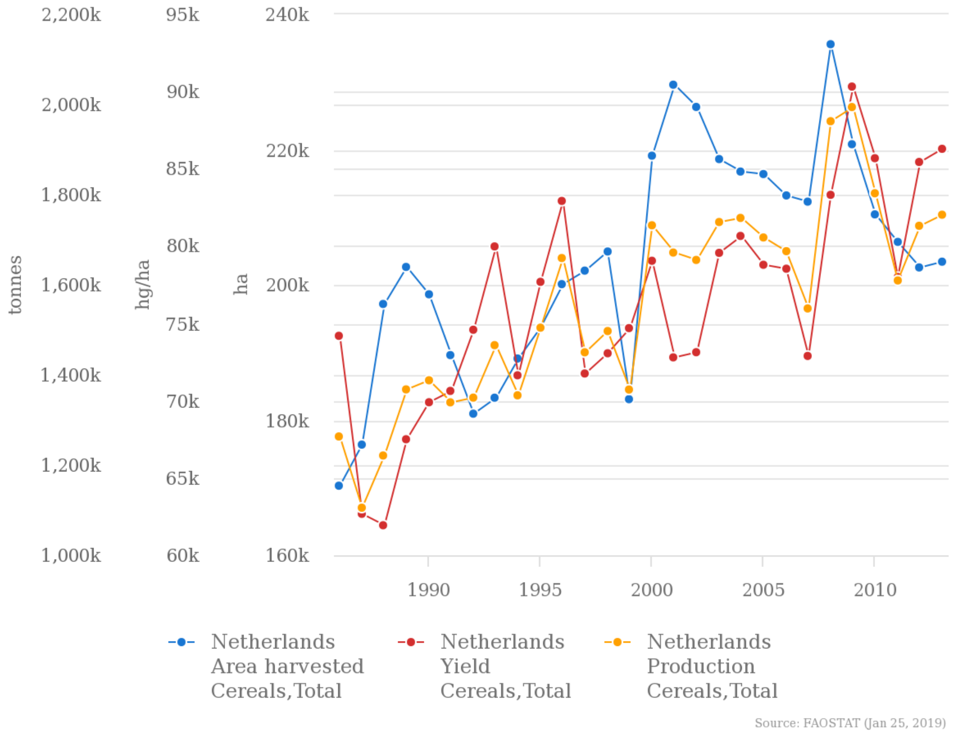

Figure N4. **Cereal production of Netherlands.**

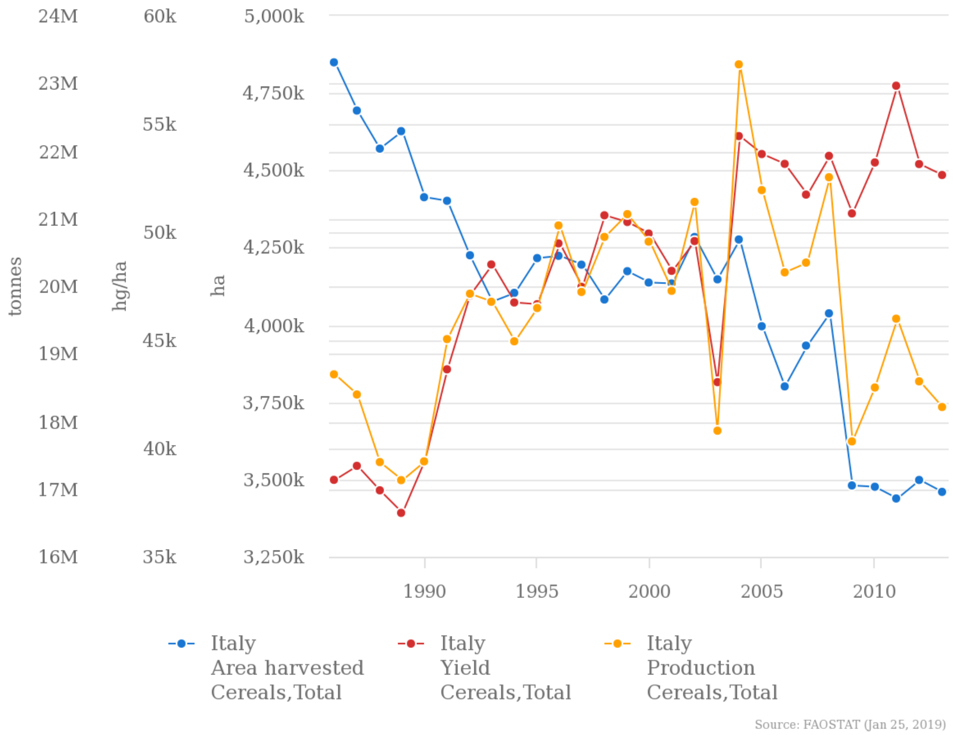

Figure N5. **Cereal production of Italy.**

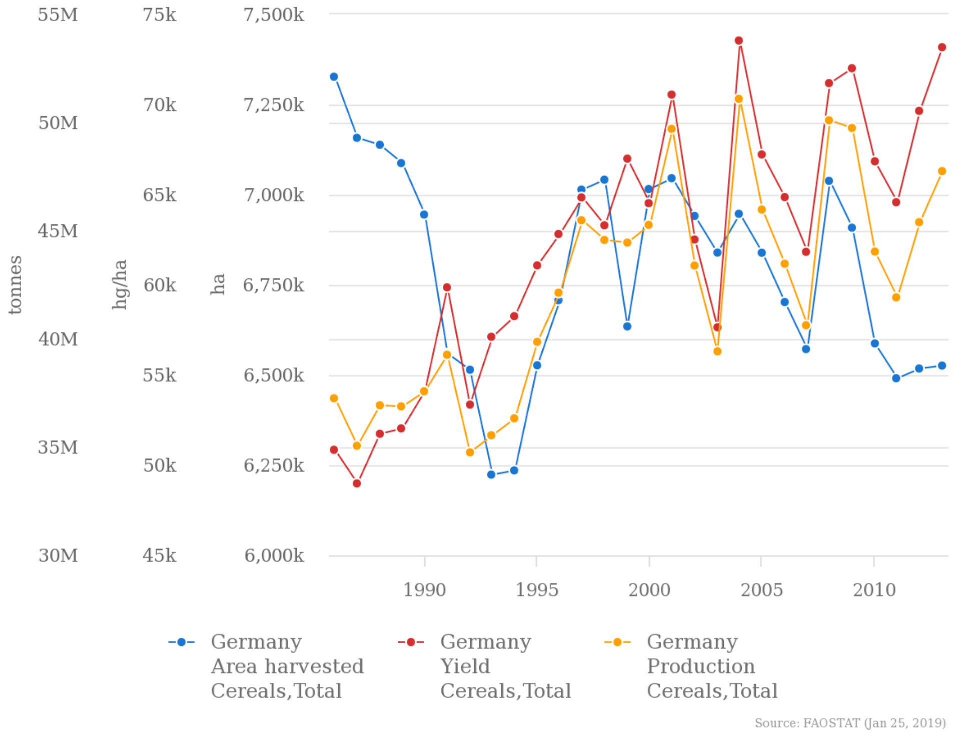

Figure N6. Cereal production of Germany.

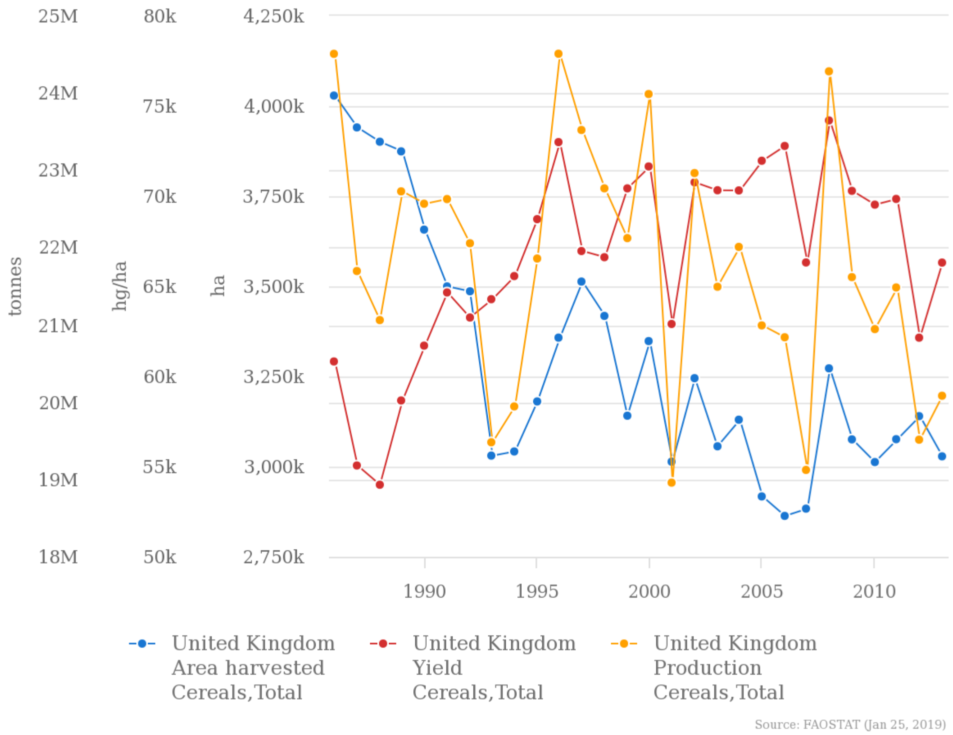

Figure N7. Cereal production of United Kingdom.

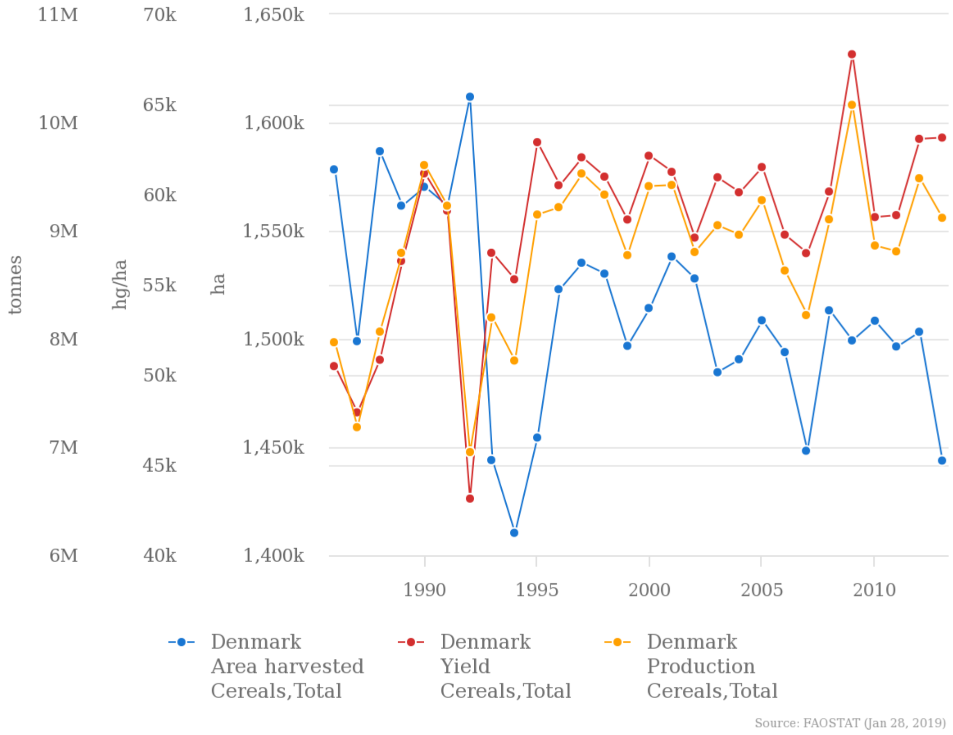

Figure N8. **Cereal production of Denmark.**

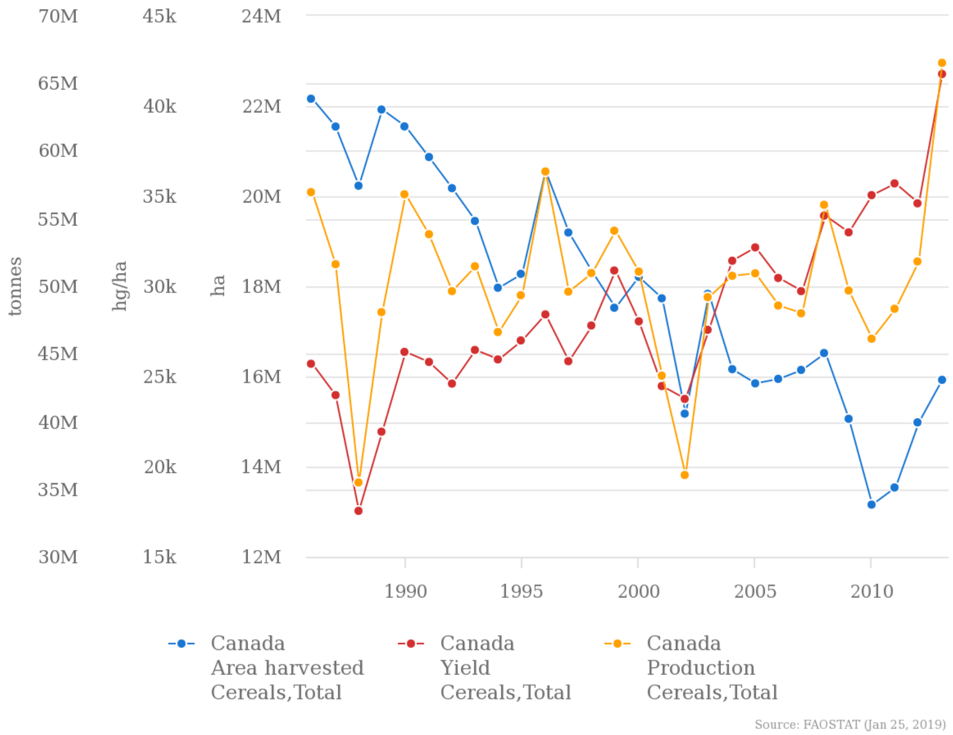

Figure N9. **Cereal production of Canada.**

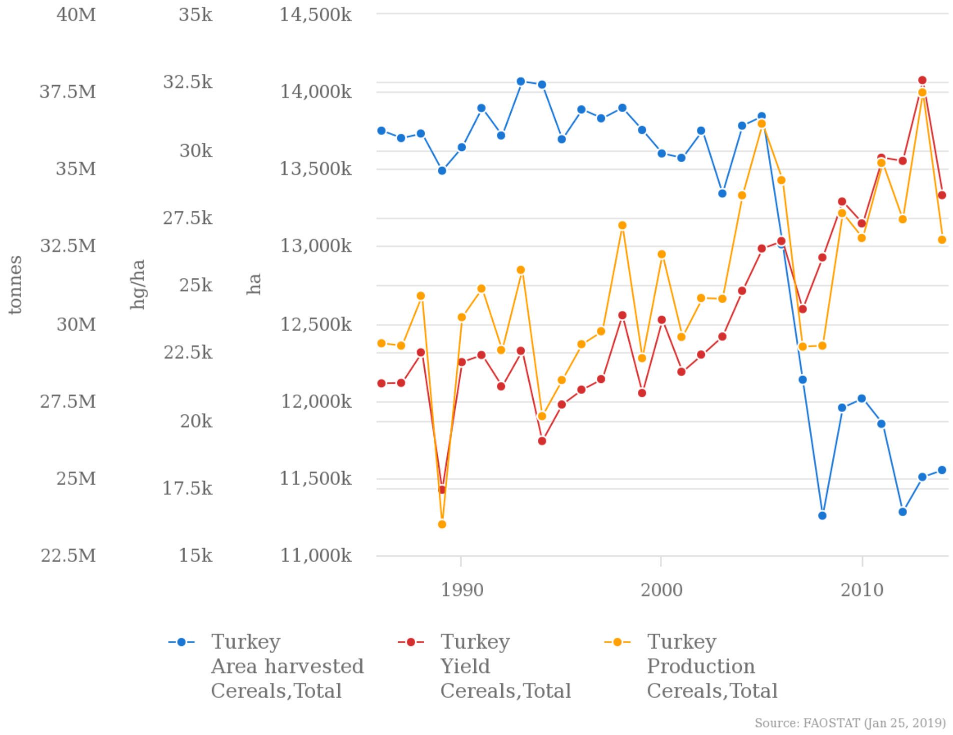

Figure N10. **Cereal production of Turkey.**

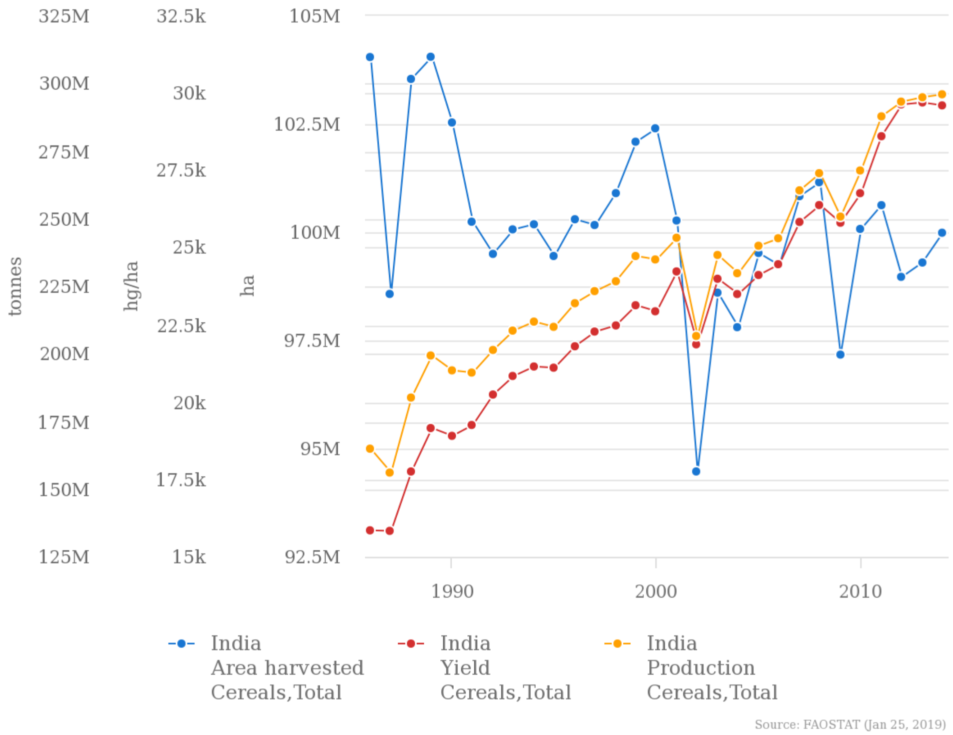

Figure N11. **Cereal production of India.**

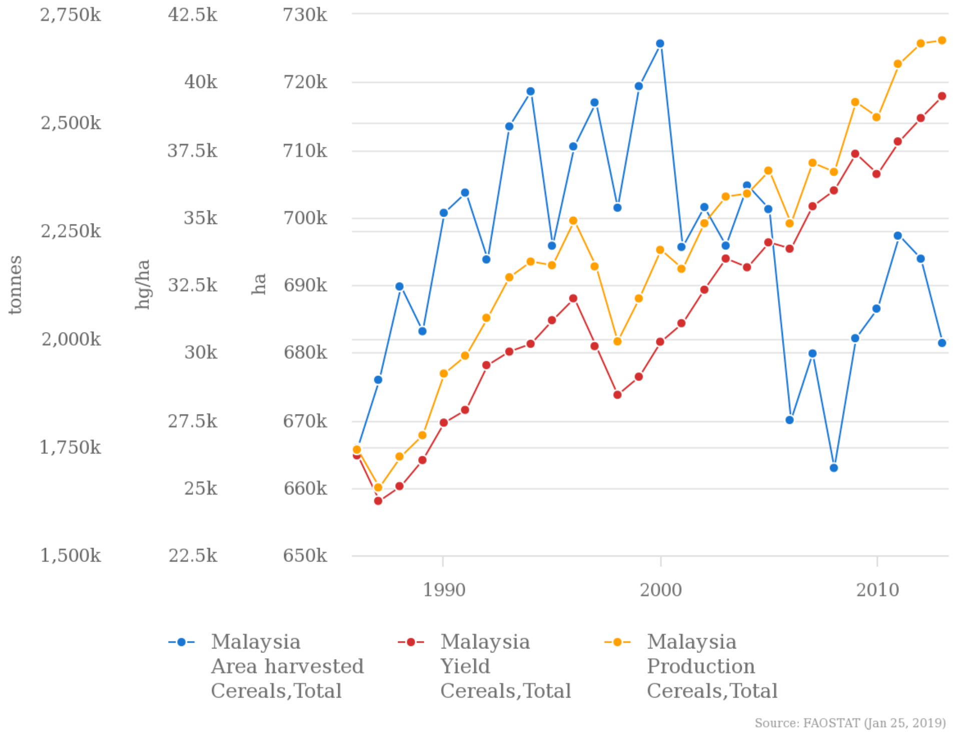

Figure N12. **Cereal production of Malaysia.**

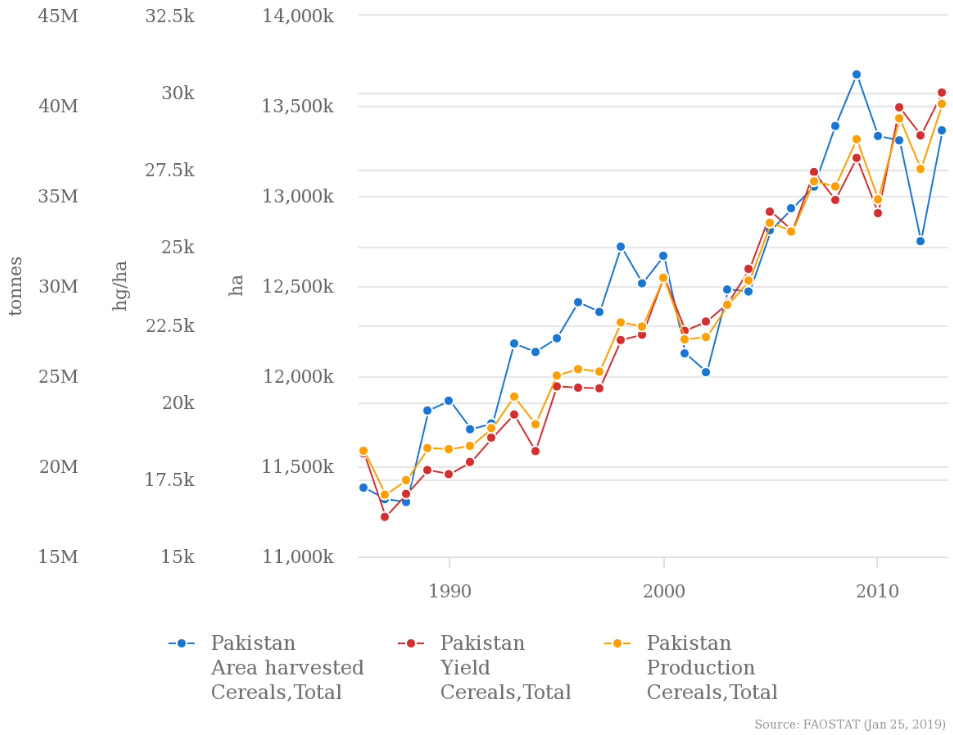

Figure N13. **Cereal production of Pakistan.**

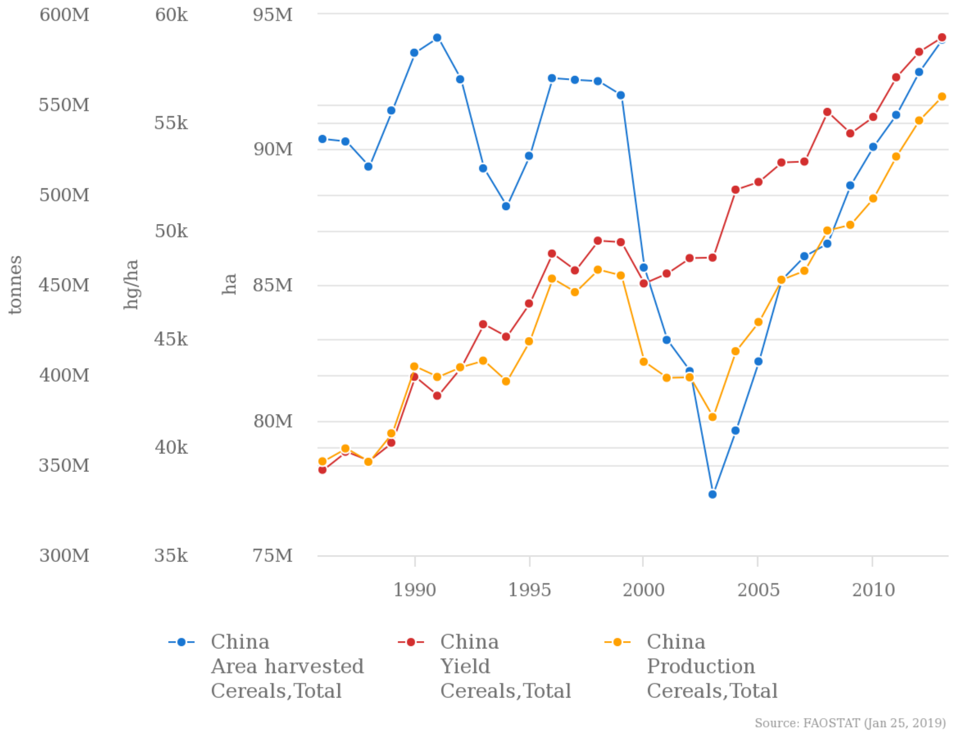

Figure N14. **Cereal production of China.**

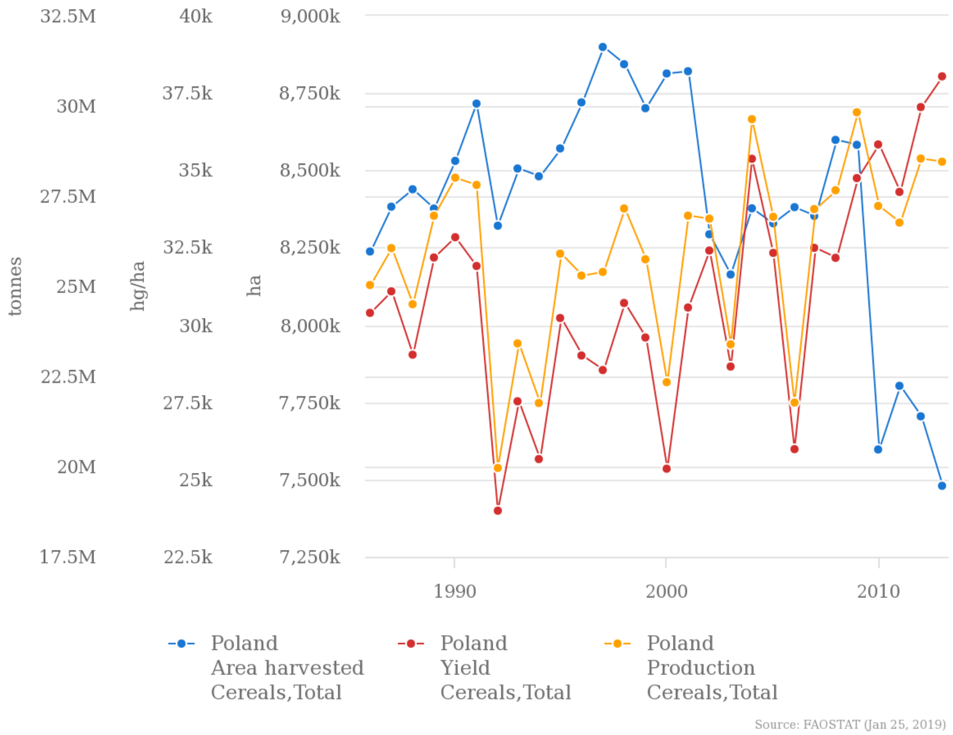

Figure N15. **Cereal production of Poland.**

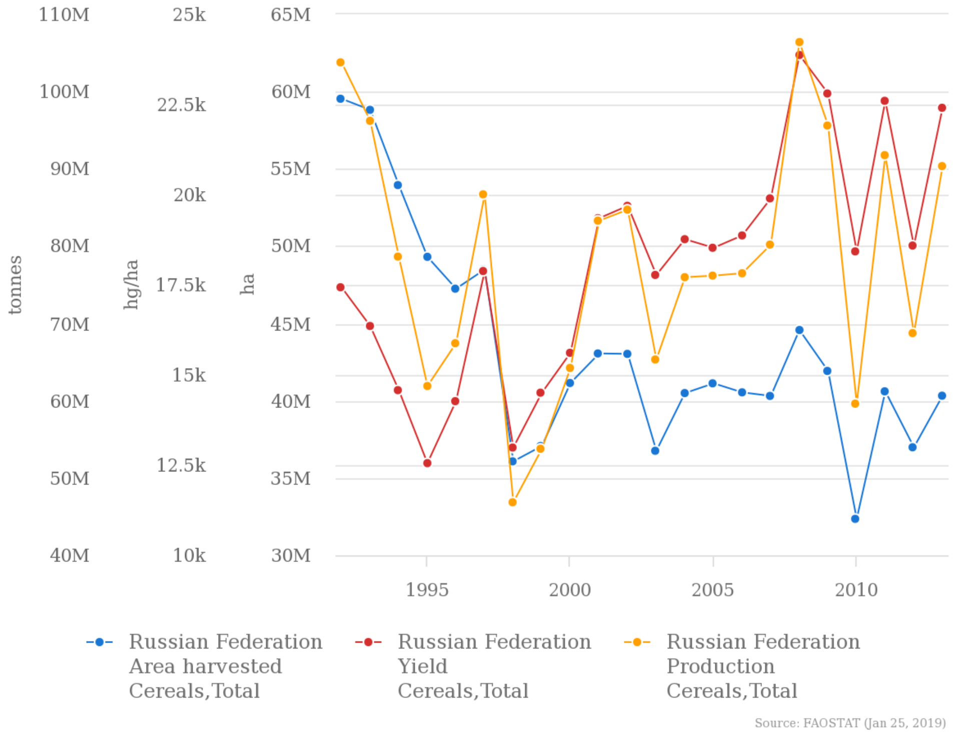

Figure N16. Cereal production of Russia.

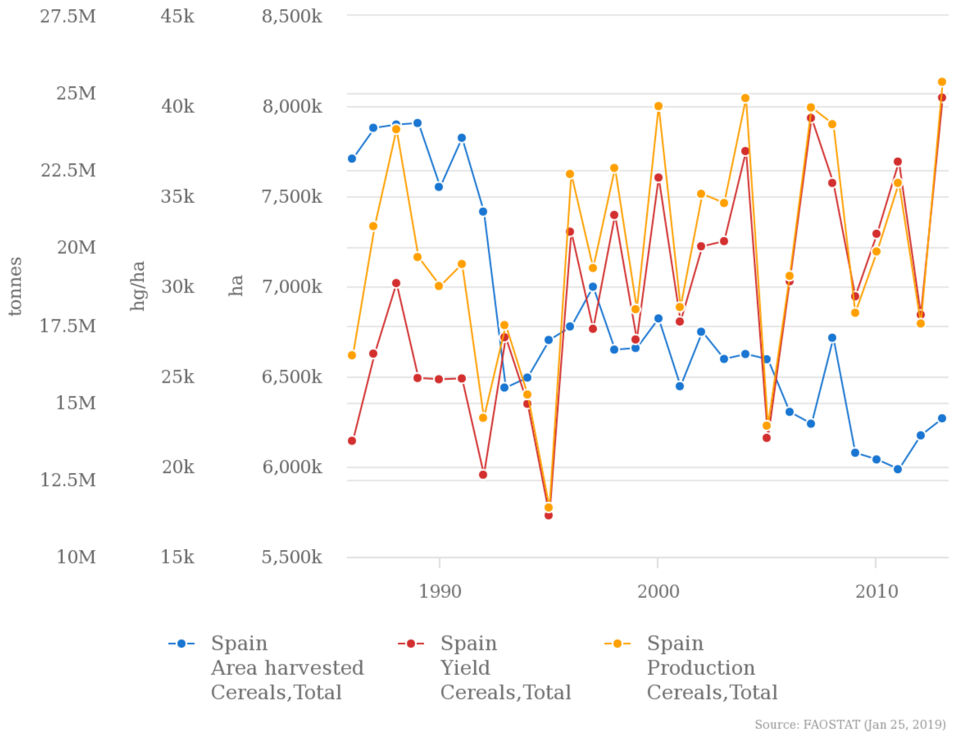

Figure N17. Cereal production of Spain.

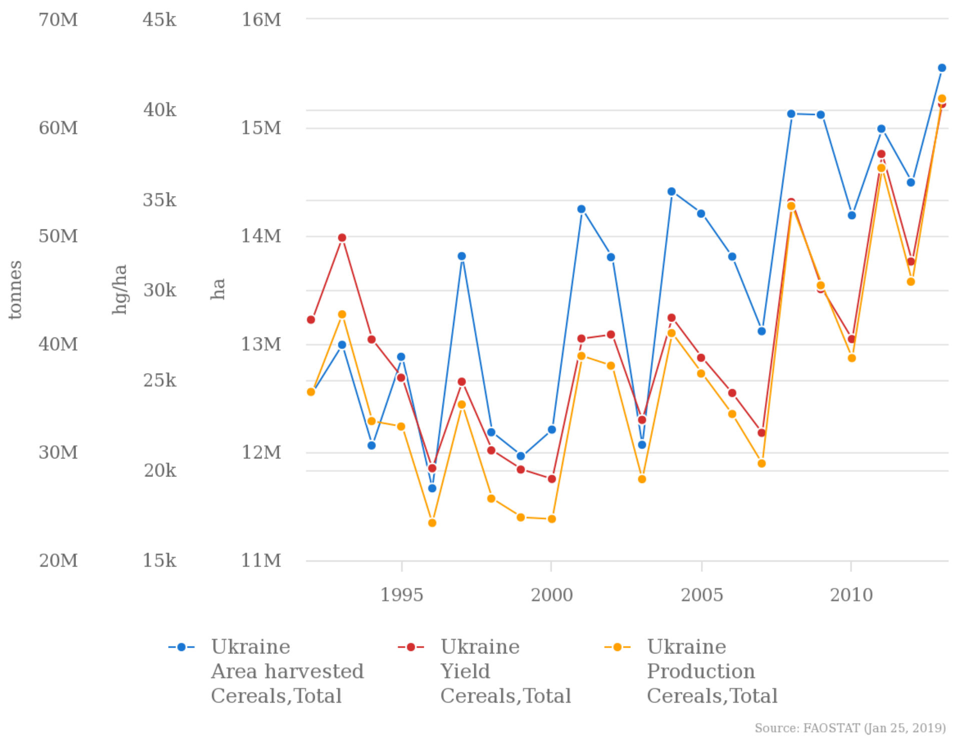

Figure N18. **Cereal production of Ukraine.**

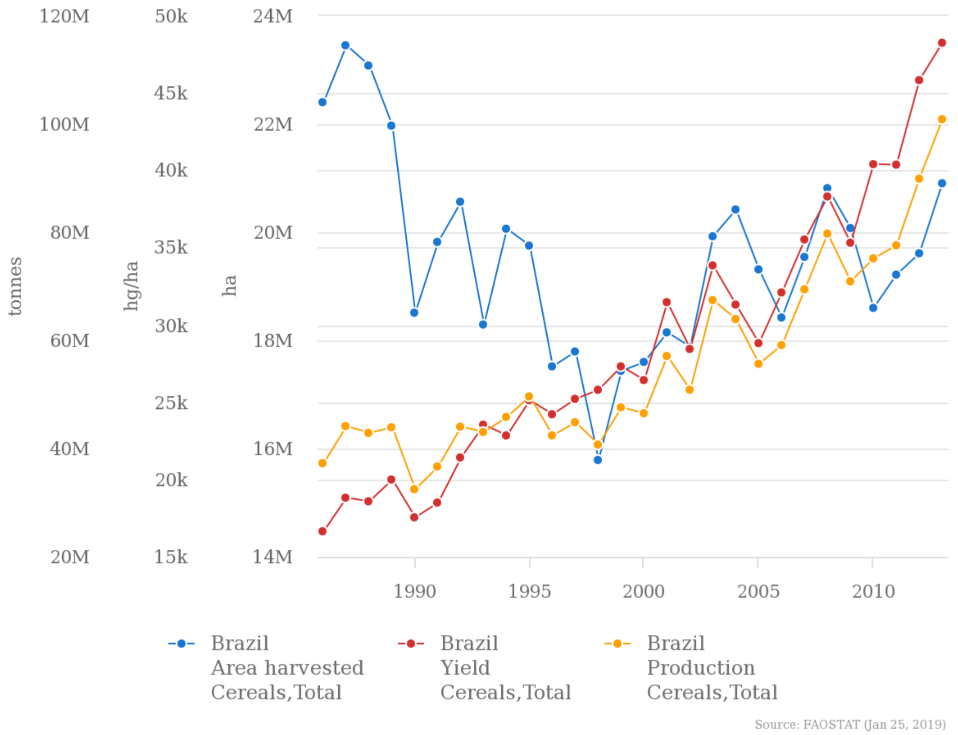

Figure N19. **Cereal production of Brazil.**

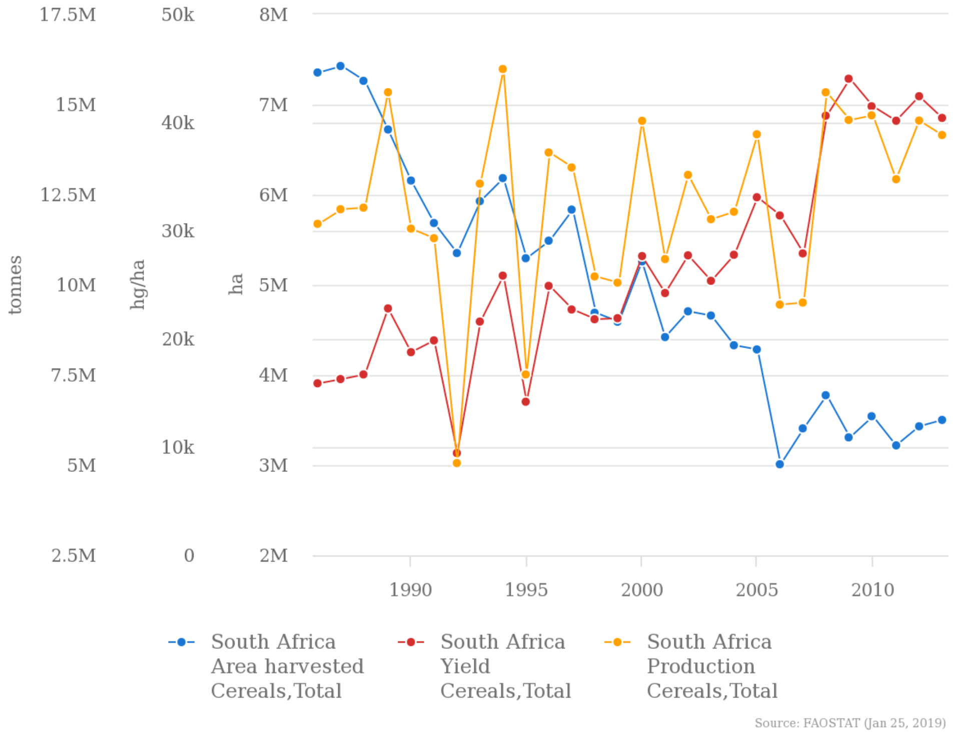

Figure N20. **Cereal production of South Africa.**

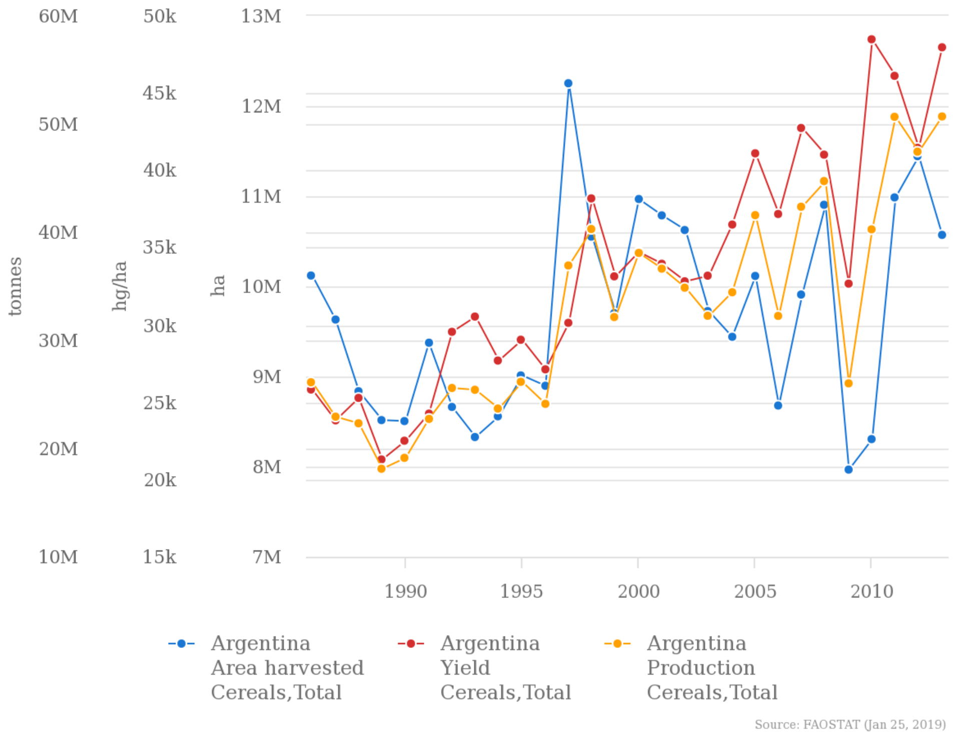

Figure N21. **Cereal production of Argentina.**

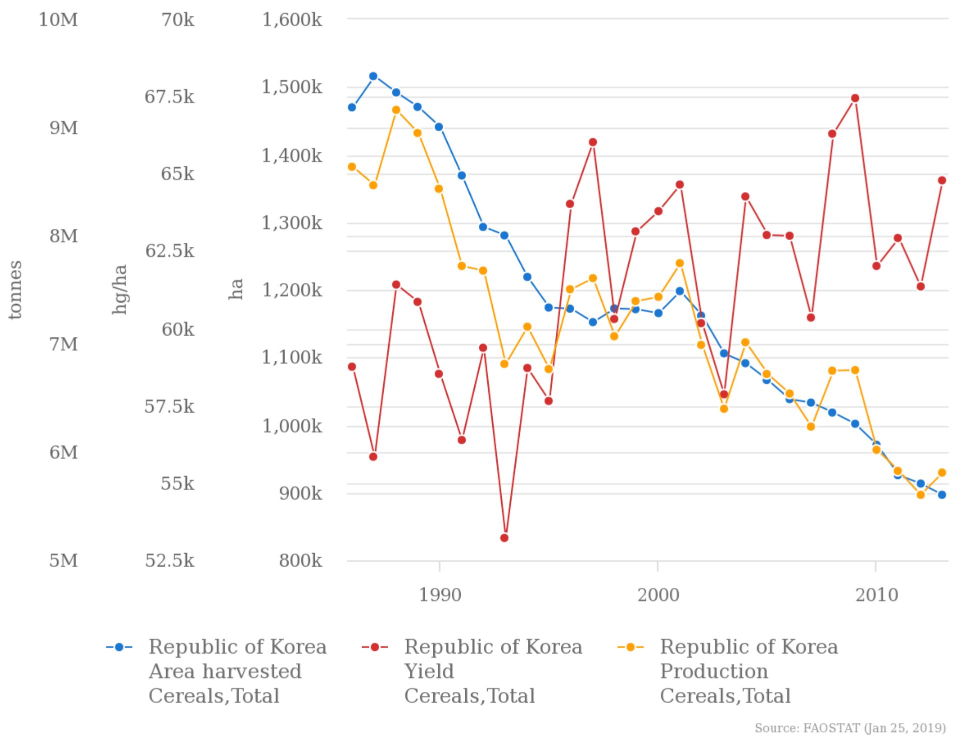

Figure N22. Cereal production of Republic of Korea.

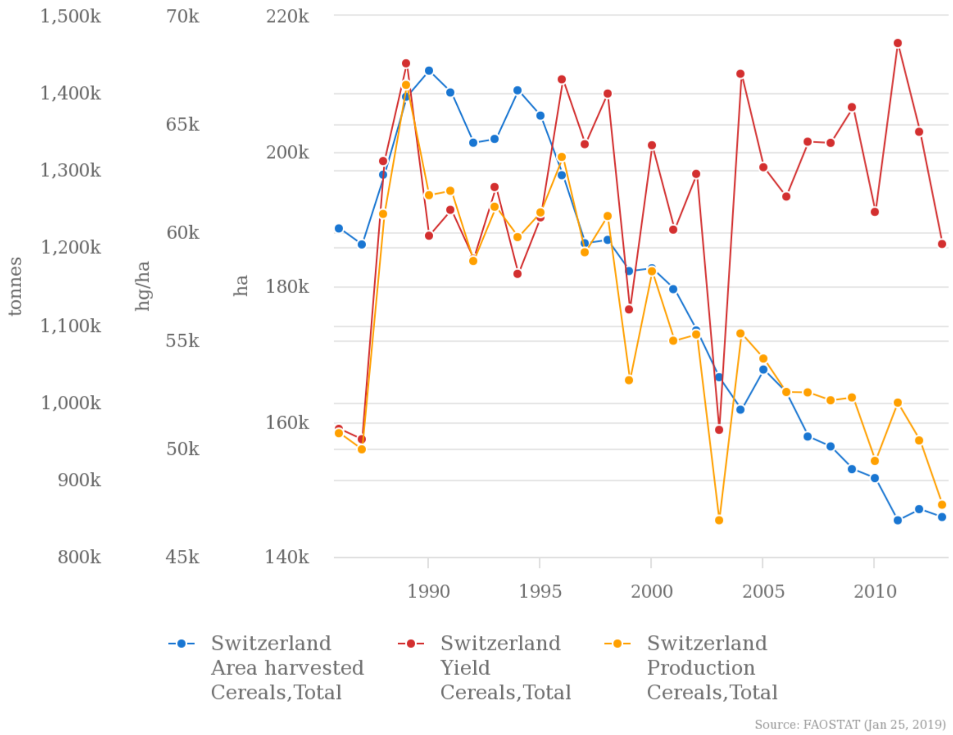

Figure N23. Cereal production of Switzerland.

Bibliography

References

1. FAO trade collection. http://ref.data.fao.org/collection?entryId=55187204-fc30-468d-9e71-953f3d498021. Accessed: 2018-06-26.
2. Alstott, J., Bullmore, E. & Plenz, D. powerlaw: a python package for analysis of heavy-tailed distributions. *PloS one* **9**, e85777 (2014).
